# DCTN1 Binds to TDP-43 and Regulates TDP-43 Aggregation

**DOI:** 10.3390/ijms22083985

**Published:** 2021-04-13

**Authors:** Manami Deshimaru, Mariko Kinoshita-Kawada, Kaori Kubota, Takuya Watanabe, Yasuyoshi Tanaka, Saito Hirano, Fumiyoshi Ishidate, Masaki Hiramoto, Mitsuru Ishikawa, Yoshinari Uehara, Hideyuki Okano, Shinichi Hirose, Shinsuke Fujioka, Katsunori Iwasaki, Junichi Yuasa-Kawada, Takayasu Mishima, Yoshio Tsuboi

**Affiliations:** 1 Department of Neurology, Faculty of Medicine, Fukuoka University, Fukuoka 814-0180, Japan; md170021@cis.fukuoka-u.ac.jp (M.D.); kawada2014@fukuoka-u.ac.jp (M.K.-K.); shinsuke@cis.fukuoka-u.ac.jp (S.F.); 2 Department of Neuropharmacology, Faculty of Pharmaceutical Sciences, Fukuoka University, Fukuoka 814-0180, Japan; kkubota@fukuoka-u.ac.jp (K.K.); twatanabe@fukuoka-u.ac.jp (T.W.); iwasakik@fukuoka-u.ac.jp (K.I.); 3 Research Institute for the Molecular Pathomechanisms of Epilepsy, Fukuoka University, Fukuoka 814-0180, Japan; yasutanaka@fukuoka-u.ac.jp (Y.T.); hirose@fukuoka-u.ac.jp (S.H.); 4 National Hospital Organization, Kokura Medical Center, Kitakyushu, Fukuoka 802-8533, Japan; hirano.saito.621@m.kyushu-u.ac.jp; 5 Bioanalysis Unit, iCeMS Analysis Center, Institute for Integrated Cell-Material Sciences, Kyoto University Institute for Advanced Study, Kyoto University, Kyoto 606-8501, Japan; ishidate.fumiyoshi.7r@kyoto-u.ac.jp; 6 Department of Neuroscience, The Scripps Research Institute, La Jolla, CA 92037, USA; hiramoto@scripps.edu; 7 Department of Physiology, Keio University School of Medicine, Tokyo 160-8582, Japan; ishimi@keio.jp (M.I.); hidokano@a2.keio.jp (H.O.); 8 Faculty of Sports and Health Science, Fukuoka University, Fukuoka 814-0180, Japan; ueharay@fukuoka-u.ac.jp

**Keywords:** dynactin, DCTN1, TDP-43, proteinopathy, Perry disease, Perry syndrome, ALS, nucleocytoplasmic transport, aggregates

## Abstract

A common pathological hallmark of several neurodegenerative diseases, including amyotrophic lateral sclerosis, is cytoplasmic mislocalization and aggregation of nuclear RNA-binding protein TDP-43. Perry disease, which displays inherited atypical parkinsonism, is a type of TDP-43 proteinopathy. The causative gene *DCTN1* encodes the largest subunit of the dynactin complex. Dynactin associates with the microtubule-based motor cytoplasmic dynein and is required for dynein-mediated long-distance retrograde transport. Perry disease-linked missense mutations (e.g., p.G71A) reside within the CAP-Gly domain and impair the microtubule-binding abilities of DCTN1. However, molecular mechanisms by which such *DCTN1* mutations cause TDP-43 proteinopathy remain unclear. We found that DCTN1 bound to TDP-43. Biochemical analysis using a panel of truncated mutants revealed that the DCTN1 CAP-Gly-basic supradomain, dynactin domain, and C-terminal region interacted with TDP-43, preferentially through its C-terminal region. Remarkably, the p.G71A mutation affected the TDP-43-interacting ability of DCTN1. Overexpression of DCTN1^G71A^, the dynactin-domain fragment, or C-terminal fragment, but not the CAP-Gly-basic fragment, induced cytoplasmic mislocalization and aggregation of TDP-43, suggesting functional modularity among TDP-43-interacting domains of DCTN1. We thus identified DCTN1 as a new player in TDP-43 cytoplasmic-nuclear transport, and showed that dysregulation of DCTN1-TDP-43 interactions triggers mislocalization and aggregation of TDP-43, thus providing insights into the pathological mechanisms of Perry disease and other TDP-43 proteinopathies.

## 1. Introduction

Dynactin, a megadalton-sized multimeric complex, acts as an essential cofactor of the microtubule-based motor cytoplasmic dynein-1 (referred to as dynein hereafter; also a megadalton-sized complex) in many eukaryotic cells [1,2]. Dynactin directly interacts with dynein and regulates its motility [3,4,5,6,7,8]. The molecular mechanisms by which dynactin controls dynein function are under intense investigation. In cells, dynein and dynactin form the major motor machinery to drive the retrograde transport of cargoes [9,10]. Dynactin subunit 1 (DCTN1)/p150^Glued^ is the largest component of dynactin [1,2,3,4]. DCTN1 binds to the dynein intermediate chain, and is both required, and is sufficient for dynactin to control dynein motility [5,6]. The other dynactin subunits play diverse regulatory roles, making dynactin a multitasking hub suitable for controlling intracellular transport. Importantly, mammalian dynein–dynactin machinery exerts processive motility required for the long-distance transport along microtubules, only when dynein and dynactin interact with a cargo adaptor (activating adaptor) to form a tripartite supercomplex [1,2,9,10].

**Figure 1:**
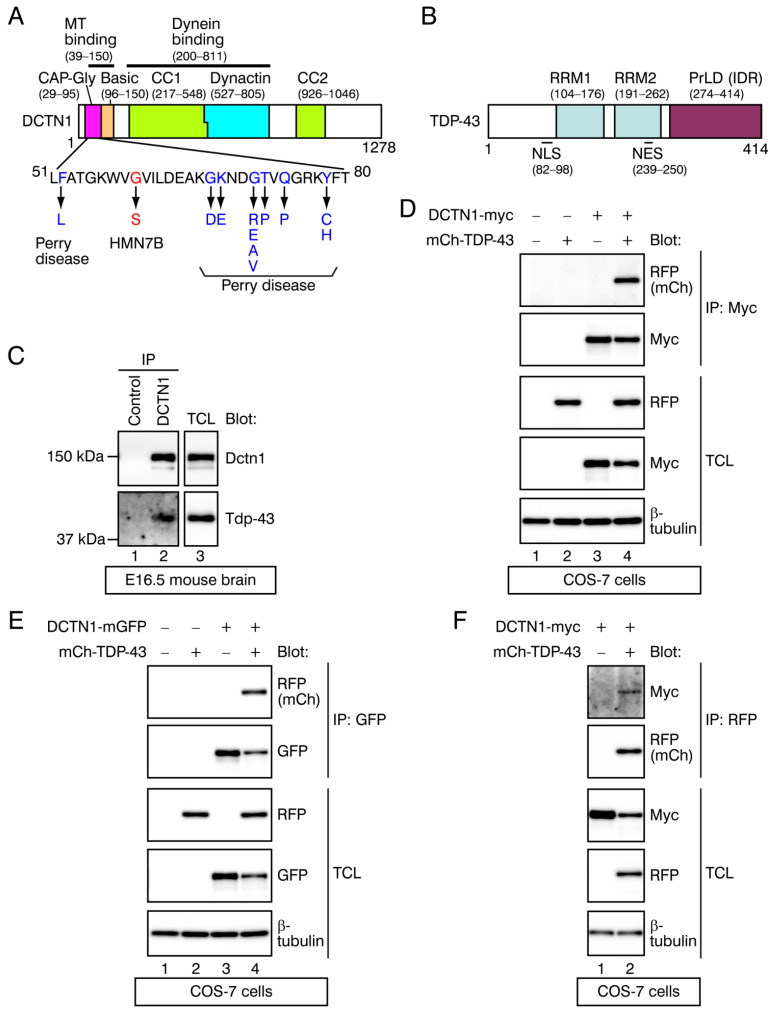
Interactions between DCTN1 and TDP-43. (**A**,**B**) Schematic diagrams of DCTN1 (**A**) and TDP-43. (**B**). In (**A**), disease-linked mutations within the DCTN1 CAP-Gly domain are indicated. In (**B**), the domain architecture of TDP-43 was modified from Winton et al. [20] and Guo et al. [21]. MT: microtubule. (**C**) Detection of interactions of endogenous Dctn1 and Tdp-43 proteins in E16.5 mouse brains. Whole brain lysates were immunoprecipitated with a control IgG or an anti-DCTN1 antibody. The immunoprecipitates and lysates were probed with antibodies as indicated in Western blots. IP: antibody used for immunoprecipitation; Blot: antibodies used for Western blotting. TCL: total cell lysates. (**D**,**E**) Interactions between DCTN1-myc (**D**) or DCTN1-mGFP (**E**) and mCherry-TDP-43 (mCh-TDP-43) proteins expressed in COS-7 cells were detected by coimmunoprecipitation. Tagged DCTN1 was immunoprecipitated using anti-myc or anti-GFP, and mCherry-TDP-43 in the immunoprecipitates was detected using anti-RFP. (**F**) Coimmunoprecipitation of DCTN1-myc and mCherry-TDP-43 in COS-7 cells in the reverse direction, relative to (**D**); mCherry-TDP-43 was immunoprecipitated using anti-RFP, and DCTN1-myc in the immunoprecipitates was detected using anti-myc.

Interactions of dynein and dynactin, including those of DCTN1, with various partner proteins have been identified through the efforts of many investigators. DCTN1 directly interacts with microtubules in a dynein-independent manner and accumulates at microtubule plus-ends [11,12]. DCTN1 is essential for the initiation of dynein-dependent retrograde transport from microtubule plus-ends at presynaptic termini [13,14]. The N-terminal cytoskeleton-associated protein glycine-rich (CAP-Gly) domain and the adjacent basic domain of DCTN1 (see Figure 1A) are responsible for microtubule binding and for dynein recruitment to microtubule plus-ends, thereby facilitating the initiation of retrograde transport from distal axons [11,12,13,14,15]. Additionally, DCTN1 regulates microtubule stability as a microtubule plus-end-tracking protein (+TIP) and as an anticatastrophe factor in neurons [16]. Because DCTN1 associates with other +TIPs, such as EB1/3 and CLIP-170, DCTN1 and these +TIPs are suspected to collaboratively regulate microtubule dynamics and capture dynamic microtubule plus-ends for initiating retrograde transport [13,17,18,19].

Dynein also binds to Huntingtin (HTT), the causative protein for Huntington’s disease (HD), while DCTN1 binds to HTT-associated protein 1 (HAP1) [22,23,24]. The HTT-HAP1 pathway physiologically regulates bidirectional trafficking along microtubules [25]. Polyglutamine repeat–expanded mutant HTT disrupts retrograde transport of autophagosomes in axons and autophagic clearance of damaged organelles [26]. Of note, knockdown of dynein or DCTN1 impairs autophagosome transport and causes motor neuron degeneration [27].

Transactive response DNA-binding protein 43 (TDP-43), encoded by the *TARDBP* gene, is a ubiquitously expressed RNA-binding protein; it is predominantly nuclear but undergoes nucleocytoplasmic shuttling. Physiologically, TDP-43 coordinates multiple aspects of RNA metabolism, for example, regulating gene transcription and RNA splicing in the nucleus, and RNA transport and protein translation in the cytoplasm and axoplasm [28,29]. TDP-43 contains two RNA recognition motifs (RRMs) and a C-terminal prion-like domain (PrLD), which is a subclass of intrinsically disordered regions (IDRs), as well as both a nuclear localization signal (NLS) and a nuclear export signal (NES), which confer the nucleocytoplasmic shuttling ability (Figure 1B) [30]. Evidence has shown that TDP-43 is intrinsically aggregation-prone, due to its PrLD/IDR [31,32]. PrLDs/IDRs have low amino acid sequence complexity, and exhibit conformational heterogeneity and thus “disordered” properties. Strikingly, 22% of disease mutations in humans are found in sequences encoding PrLDs/IDRs [33]. PrLD/IDR-containing RNA-binding proteins, such as TDP-43, mediate protein/RNA interactions to form membraneless organelles, via liquid–liquid phase separation (LLPS) into a dense phase and a dilute phase [32]. Such PrLD/IDR-harboring molecules are able to switch between monomeric dispersed states and multimeric condensed liquid states. Phase-separated liquid droplets may even mature to hydrogels and fibrillar aggregates, over time [32,34]. Recent studies have revealed that multivalent, intermolecular contacts control these phase transition processes [32].

In 2006, almost simultaneously, two research groups reported TDP-43 to be a major ubiquitinated protein component of insoluble cytoplasmic aggregates in degenerating neurons found in patients with frontotemporal dementia (FTD) and a fatal motor neuron disease, amyotrophic lateral sclerosis (ALS) [35,36]. These studies raised the hypothesis that FTD and ALS shared a common neuropathological mechanism: TDP-43 proteinopathy [34,37]. Consistently, missense mutations in TDP-43, which mainly distribute to its C-terminal PrLD, were found to be causative of familial ALS (designated *ALS10*) and sporadic ALS; expression of mutant TDP-43 induces neural apoptosis [38,39]. The ALS-linked TDP-43 mutations accelerate aberrant aggregation processes, thereby increasing cell toxicity [31,32,39]. Thus, the concept of TDP-43 as a disease protein has been established. The cytoplasmic aggregates of TDP-43 in diseased neurons have been detected in not only the ALS/FTD spectrum but also several other neurodegenerative diseases [40,41]. However, molecular mechanisms that cause TDP-43 cytoplasmic mislocalization and aggregation remain unsolved.

Dysfunction of the dynein–dynactin retrograde motor machinery has been implicated in the pathogenesis of several neurodegenerative diseases [42,43]. With regard to *DCTN1*, closely apposed mutations in its CAP-Gly domain cause two distinct neurodegenerative diseases (Figure 1A): distal hereditary motor neuropathy 7B (HMN7B) and Perry disease (Perry syndrome). HMN7N is an ALS-like motor neuron disease, caused by a p.G59S missense mutation [44]. On the other hand, Perry disease is an autosomal dominant, fatal neurodegenerative disease characterized clinically by early-onset parkinsonism, depression/apathy, weight loss and central hypoventilation; and pathologically by degeneration of substantia nigral dopaminergic neurons and TDP-43 proteinopathy [45,46,47,48,49,50]. Eleven missense, Perry disease-linked mutations in *DCTN1* (e.g., p.G71A) have been identified so far (Figure 1A) [49,51]. Although these DCTN1 mutants exhibit impairments of CAP-Gly domain function, such as microtubule-binding and retrograde transport initiation [13,14,44,47], pathological mechanisms that distinctly cause Perry disease or HMN7B are poorly understood. In autopsy studies, TDP-43 proteinopathy has been detected in patients with Perry disease, but not in those with HMN7B [47,48,50]. These observations suggest that mutation positions in *DCTN1* differentially affect TDP-43 aggregation, although the underlying mechanisms are unknown.

The first goal of this study was to characterize the biochemical relationship between DCTN1 and TDP-43. To investigate the molecular basis of Perry disease, we have recently been searching for DCTN1-interacting proteins involved in neurodegeneration. However, no novel, promising candidates for such DCTN1 interactors were found. We thus refocused on the findings that, in our immunohistochemical studies, both TDP-43 proteinopathy and dynactin aggregates were detected in all of the Perry disease post-mortem brains examined [47,48,50]. Relatedly, genetic interactions between a Perry disease-linked mutant *DCTN1* and a TDP-43 ortholog in *Drosophila* were recently revealed [52]. These findings prompted us to address the simple question of whether DCTN1 physically associates with TDP-43; further predicting a new hypothesis that, if so, the abnormality in these interactions compromises TDP-43 distribution in the nucleus. Through coimmunoprecipitation and in vitro pull-down experiments, we demonstrated that DCTN1 binds to TDP-43. Furthermore, we present evidence that DCTN1 is involved in regulating TDP-43 cytoplasmic-nuclear transport and aggregation: DCTN1^G71A^ or truncated mutant DCTN1 induced the processes of cytoplasmic mislocalization and aggregation of TDP-43 in non-neuronal cells and induced pluripotent stem cell (iPSC)-derived neurons, thereby recapitulating several cellular phenotypes found in the brain neurons of Perry disease patients.

## 2. Results

### 2.1. Identification of TDP-43 as a DCTN1-Interacting Protein

To examine whether DCTN1 interacted with TDP-43, we performed coimmunoprecipitation between endogenous Dctn1 and Tdp-43 proteins in murine brains. We prepared whole brain extracts from embryonic day (E) 16.5 mice. An anti-DCTN1 antibody was used to immunoprecipitate Dctn1 from the brain cell lysates, and an anti-TDP-43 antibody was used to detect Tdp-43 in the immunoprecipitates through Western blotting. Results of our coimmunoprecipitation experiment revealed that the endogenous Dctn1 and Tdp-43 interacted with each other in vivo (Figure 1C).

To confirm the potential interactions between DCTN1 and TDP-43, we next performed coimmunoprecipitation between tagged DCTN1 and TDP-43 proteins in cultured cells. cDNAs encoding the full-length human DCTN1 C-terminally tagged with 2× myc epitope (DCTN1-myc) or with monomeric green fluorescent protein (DCTN1-mGFP) and/or full-length human TDP-43 N-terminally tagged with mCherry (monomeric red fluorescent protein (mRFP) derivative; mCherry-TDP-43) were transfected into monkey fibroblast COS-7 cells. DCTN1-myc or DCTN1-mGFP were immunoprecipitated using an anti-myc or anti-GFP antibody, respectively, from the cell lysates (Figure 1D,E). In both experimental settings, the full-length mCherry-TDP-43 was detected by an anti-RFP antibody, only in those precipitates from lysates of cells expressing both the tagged DCTN1 and TDP-43 (Figure 1D,E, lane 4 each; see the top panels). We next carried out coimmunoprecipitation in the reverse direction: upon immunoprecipitating mCherry-TDP-43 using anti-RFP, DCTN1-myc was detected only in those precipitates from lysates of cells expressing both the tagged DCTN1 and TDP-43 (Figure 1F, lane 2). Collectively, the reciprocal coimmunoprecipitation between DCTN1 and TDP-43 confirmed that DCTN1 formed complexes with TDP-43.

### 2.2. Determination of the Interacting Regions within DCTN1 and TDP-43

We sought to map the TDP-43-interacting domains of DCTN1. A series of truncated mutants of DCTN1-myc were made and subjected to coimmunoprecipitation assays. Aiming at high-precision domain dissection, bioinformatics analyses were performed on the amino acid sequence of DCTN1 (1,278 amino acids (aa)) using online resources, including SMART-EMBL Heidelberg [53], Multicoil2 (coiled-coil (CC) region-detecting algorithm) [54], PONDR (predictor of naturally disordered regions) [55], and NCPR (net charge per residue) [56]. DCTN1 contains the CAP-Gly and the immediately following basic domains and the two CC domains (CC1 and CC2) [1,2,15] (Figure 1A). Furthermore, five putative IDRs were detected by PONDR (Figure S1A). CC1 corresponds to a disordered CC with two clusters of acidic regions, while CC2 corresponds to a disordered CC with a uniform charge distribution [57]. A previously undocumented domain unique to DCTN1 was recognized and designated as the dynactin domain, according to SMART-EMBL (Figure 1A and Figure S1A, blue bar). The dynactin domain lacks an obviously characteristic region, according to PONDR and NCPR, except for a short cluster sequence at its C-terminus (marked with asterisks in Figure S1A). We adopted the positions of CCs as described by Schroer [1] and King et al. [58], as well as SMART-EMBL: CC1 and CC2 located to 217–548 and 926–1046 aa, respectively. CCs are frequently found to mediate interactions between components in the dynein–dynactin-adaptor complex [1]. The microtubule-binding region (39–150 aa) corresponds to the CAP-Gly-basic supradomain, while the dynein-binding region resides at 200–811 aa, spanning the CC1 and dynactin domains (Figure S1A) [5,6,11]. On the other hand, TDP-43 contains a well-known PrLD (IDR) with a few charge distributions in the C-terminal region [31,32,34], but no obvious CC regions (Figure S1B).

**Figure 2:**
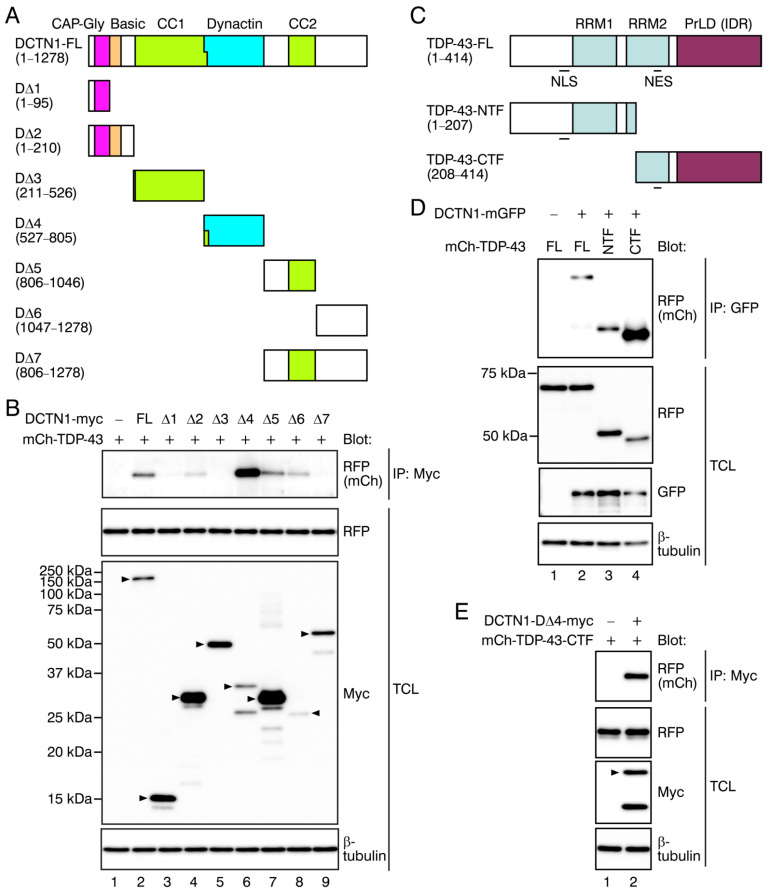
The interacting regions of DCTN1 and TDP-43. (**A**) Schematic representations of a series of truncated forms of mutant DCTN1 used in this study. FL: full-length. (**B**) Coimmunoprecipitation (co-IP) of DCTN1-myc mutants with mCherry-FL TDP-43 in COS-7 cells. Immunoprecipitation and Western blotting were performed using the antibodies indicated. Arrowheads in the Western blots indicate the expected positions of tagged DCTN1 fragments. (**C**) Schematic representations of the N-terminal fragment (NTF) and C-terminal fragment (CTF) of TDP-43. (**D**) Coimmunoprecipitation between FL DCTN1-mGFP and mCherry-TDP-43-NTF or CTF. (**E**) Coimmunoprecipitation between myc-tagged DCTN1 D∆4 fragment and mCherry-tagged TDP-43-CTF.

Based on these results, we divided the DCTN1-coding sequence to create expression plasmids encoding seven truncated mutant forms of DCTN1-myc, as illustrated in Figure 2A. We generated two types of N-terminal fragments of DCTN1: D∆1 (1–95 aa) contains the N-terminal end and CAP-Gly domain, while D∆2 (1–210 aa) contains the N-terminal end, the CAP-Gly domain, and the basic domain (D∆2 is equivalent with the DCTN1 N-terminal fragment used previously [16]). D∆3 holds the CC1 domain alone, while D∆4 holds the dynactin domain alone. Furthermore, according to the position of CC2, the C-terminal region (806–1278 aa, corresponding to D∆7) was divided into D∆5 and D∆6.

These truncated mutant DCTN1-myc and mCherry-tagged full-length TDP-43 were cotransfected into COS-7 cells, and their interactions were examined by coimmunoprecipitation. TDP-43 was detected in the anti-myc immunoprecipitates from cells expressing full-length DCTN1 or several mutant forms; the CAP-Gly-basic supradomain-containing fragment (D∆2), dynactin domain (D∆4), CC2-containing region (D∆5), and extreme C-terminal region (D∆6) of DCTN1 interacted with TDP-43 (Figure 2B, lanes 4, 6, 7, and 8; see the top panel). The CAP-Gly domain alone (D∆1) only faintly interacted with TDP-43 (lane 3). On the other hand, the CC1 domain (D∆3) and the whole C-terminal region subsequent to the dynactin domain (D∆7) exhibited no detectable interaction with TDP-43 (Figure 2B, lanes 5 and 9). Altogether, DCTN1 interacted with TDP-43 via not only the CAP-Gly-basic domain but also via the dynactin domain, CC2 domain-containing region, and extreme C-terminal region, indicating the multivalency of DCTN1-TDP-43 interactions.

We next divided the TDP-43-coding sequence into two parts, to express the N-terminal fragment (NTF; 1–207 aa) or the PrLD-containing C-terminal fragment (CTF; 208–414 aa; Figure 2C). This Arg208-C-terminus fragment corresponds to a pathological TDP-43-CTF, which was identified as a major ubiquitinated and hyperphosphorylated component of cytoplasmic aggregates in ALS/FTD patient brains, generated by proteolytic cleavage [59]. Our CTF construct was made with the same design as the previously used plasmid encoding the pathologically active mutant [59,60]. DCTN1 interactions of the TDP-43-CTF were much stronger than those of the full-length TDP-43 or NTF (Figure 2D), indicating that the C-terminal region of TDP-43 preferentially contributed to DCTN1 interactions.

Furthermore, the DCTN1 dynactin domain (D∆4) and the TDP-43-CTF were sufficient for interactions between DCTN1 and TDP-43 (Figure 2E). Altogether, our data revealed that DCTN1 and TDP-43 interacted with each other, preferably via the CAP-Gly-basic domain, dynactin domain, and extreme C-terminal region of DCTN1, and via the TDP-43 C-terminal region.

### 2.3. Effects of Point Mutations in DCTN1 and TDP-43 on Their Interactions

**Figure 3:**
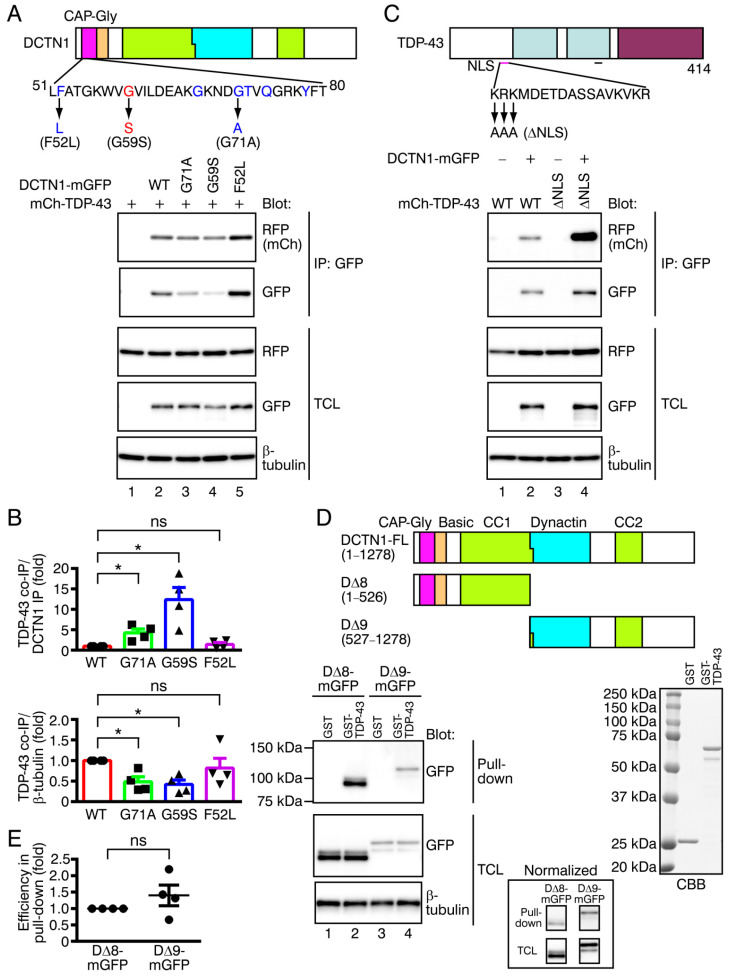
Effects of missense mutant DCTN1 and NLS-deficient mutant TDP-43 on the DCTN1-TDP-43 interactions, and in vitro DCTN1-TDP-43 binding. (**A**) Interactions of disease-linked missense mutants of DCTN1-mGFP, p.G71A (Perry disease), p.G59S (HMN7B), or p.F52L (Perry disease) with mCherry-tagged wild-type (WT) TDP-43 were examined by coimmunoprecipitation (co-IP) in COS-7 cells. (**B**) Quantification of interactions of DCTN1 missense mutants with WT TDP-43, relative to WT DCTN1. The top panel shows the ratio of mCherry-TDP-43 to DCTN1-mGFP in the immunoprecipitates, and the bottom panel shows the ratio of the immunoprecipitated mCherry-TDP-43 normalized to levels of β-tubulin in the total cell lysates (TCL). Here and in subsequent figures, data are represented as the mean ± SEM. *n* = 4 (independent experiments). * *p* < 0.05 compared to WT by two-tailed paired *t*-test. ns: not significant. (**C**) Interactions between WT DCTN1-mGFP and TDP-43-∆NLS mutant. (**D**) A GST pull-down assay between DCTN1 fragments and GST-fused WT TDP-43. Cell lysates prepared from COS-7 cells expressing truncated mutant DCTN1 (D∆8-mGFP or D∆9-mGFP, top panel) were incubated with glutathione Sepharose beads preloaded with purified GST control or GST-TDP-43 (bottom right panel). The bound DCTN1 fragment was detected by Western blotting using anti-GFP (bottom left panel). The result was normalized according to the GFP signal intensity in TCLs, and is shown in the inset. (**E**) Quantification of DCTN1 truncated mutants pulled down with GST-TDP-43. The GST-pulled-down levels of D∆9-mGFP were normalized to those of D∆8-mGFP. *n* = 4 (independent experiments). *p* = 0.2949 (two-tailed paired *t*-test).

Two different groups of missense mutations within the DCTN1 CAP-Gly domain are known to cause Perry disease, accompanying TDP-43 proteinopathy, or HMN7B, with no detectable TDP-43 proteinopathy [47,48,50]. We tested whether disease-linked missense mutations in DCTN1 affected DCTN1-TDP-43 interactions. We coexpressed wild-type or mutant DCTN1-mGFP (p.G71A (Perry disease), p.G59S (HMN7B), p.F52L (late-onset and moderate Perry disease); see Figure 3A, top panel) together with mCherry-tagged wild-type TDP-43 in COS-7 cells and performed coimmunoprecipitation (Figure 3A, bottom panels). Because expression levels and the efficacy of protein recovery in immunoprecipitation varied markedly among wild-type and mutant DCTN1, possibly due to instability and aggregation of the mutants [13,61], we used two types of quantification to examine TDP-43 interactions of mutant DCTN1. We calculated the ratio of the level of TDP-43 to that of DCTN1 detected in immunoprecipitates, following the previously used method [13] (Figure 3B, top panel). Additionally, the levels of coimmunoprecipitated TDP-43 were normalized to β-tubulin levels in cell lysates, which represented the amounts in cells (Figure 3B, bottom panel). Unexpectedly, the relative efficiency of interactions of DCTN1^G71A^ and DCTN1^G59S^ mutants with TDP-43 was significantly higher than those of wild-type DCTN1 (Figure 3B, top panel; p.G71A: 4.25 ± 0.93-fold of the wild-type level, *p* = 0.0249; p.G59S: 12.38 ± 2.99-fold, *p* = 0.0121). On the other hand, the interactions of DCTN1^F52L^ with TDP-43 were not significantly different from those of wild-type DCTN1 (TDP-43/ DCTN1 ratio: 1.43 ± 0.47-fold, *p* = 0.4304), which was consistent with clinical observations that symptoms of patients with the *DCTN1* p.F52L mutation are less severe than with the other mutation types [62]. Taken together, both p.G71A and p.G59S mutations in the CAP-Gly domain affected TDP-43 interactions of DCTN1. Perry disease-linked and HMN7B-linked mutant DCTN1 may exhibit increased preferences for interacting with TDP-43, in contrast with reduced microtubule-binding abilities.

We next examined interactions between wild-type DCTN1 and a defective NLS-carrying mutant TDP-43 (TDP-43-∆NLS). As previously reported [20], contrary to wild-type TDP-43, the major pools of TDP-43-∆NLS were excluded from the nucleus and distributed within the cytoplasm (unpublished observations). TDP-43-∆NLS showed a drastic increase in DCTN1 interactions, as compared with wild-type TDP-43 (Figure 3C), possibly reflecting a change in the predominant location of TDP-43 from the nucleus to the cytoplasm. This result indicates that DCTN1 does not interact selectively with the TDP-43 NLS, and that DCTN1 interacts with TDP-43 mainly in the cytoplasm. However, we do not exclude the possibility that they also interact with each other in the nucleus (see Discussion).

### 2.4. In Vitro Binding between DCTN1 and TDP-43

We examined whether DCTN1 bound to TDP-43 in vitro. The DCTN1-coding sequence was divided into two parts: to express the NTF composed of the CAP-Gly-basic and CC1 domains (D∆8), or the CTF composed of the dynactin and CC2 domains and extreme C-terminal region (D∆9) (Figure 3D, top panel). D∆8 or D∆9 tagged with mGFP were expressed in COS-7 cells and the cell lysates were prepared. GST-tagged full-length TDP-43 was expressed in *E. coli* and purified using glutathione Sepharose beads (Figure 3D, bottom right panel). We then performed a GST pull-down assay by incubating these D∆8 or D∆9-containing cell lysates with beads preloaded with purified GST-TDP-43. Both D∆8 and D∆9 were pulled down by GST-TDP-43 with comparable efficiencies, but not by the GST control (Figure 3D, bottom left panel and inset, E). Therefore, our data showed that DCTN1 binds to TDP-43 in cells and in vitro.

### 2.5. Effects of Disease-Linked Point Mutants and Truncated Mutants of DCTN1 on the Subcellular Localization and Aggregation of TDP-43

**Figure 4:**
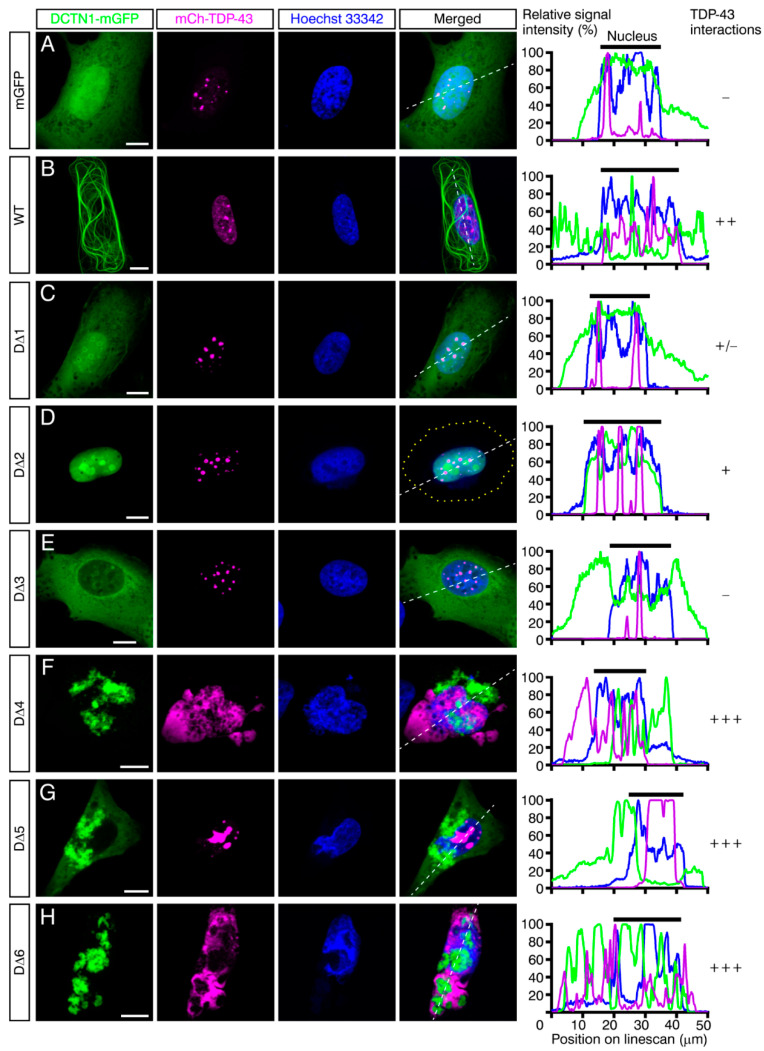
Truncated mutant forms of DCTN1 that contain the dynactin domain or C-terminal region cause TDP-43 mislocalization and aggregation. Maximum-intensity projections of deconvoluted z-stack confocal images of U2OS cells that coexpressed mGFP (**A**), wild-type (WT) (**B**) or mutant (**C–H**) DCTN1-mGFP and mCherry-tagged WT TDP-43 are shown. The transfected cells were cultured for three days under nonstressed conditions, fixed, and subjected to confocal microscopy. Scale bars, 10 μm. The graphs show linescans of the cells along the white broken lines. The intensity of interactions between mutant DCTN1 and TDP-43, based on coimmunoprecipitation in Figure 2B, is shown on the right.

**Figure 5:**
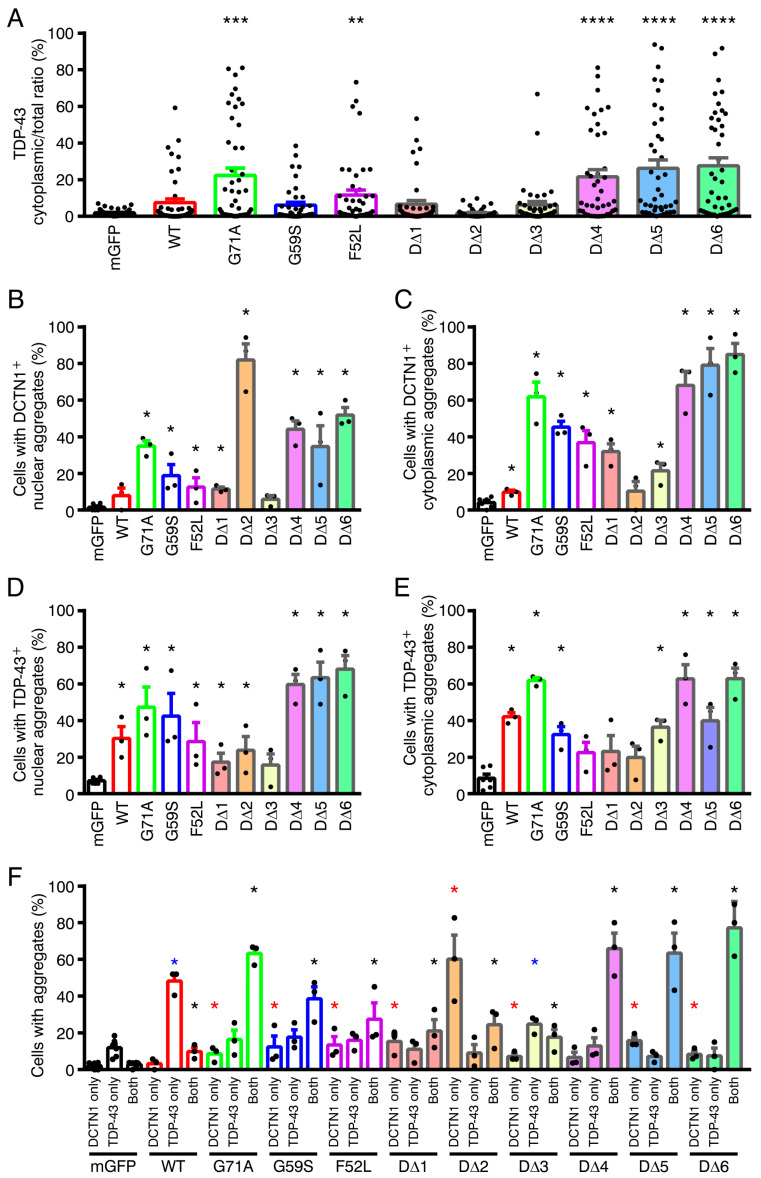
Effects of mutant DCTN1 on TDP-43 localization and aggregation of DCTN1 and TDP-43 in U2OS cells. (**A**) Quantification of cytoplasmic mislocalization of TDP-43. *n* = 45, 45, 46, 40, 44, 41, 40, 40, 41, 41, and 42 (cells) from left to right (three to four independent experiments). (**B**–**E**) The percentages of cotransfected cells with DCTN1 (**B**,**C**)- or TDP-43 (**D**,**E**)-positive aggregates in the nucleus (**B**,**D**) and cytoplasm (**C**,**E**) are shown. *n* = 6 (experiments) for the mGFP control group and *n* = 3 for the other groups (50–60 cells per experimental group). (**F**) Quantification of the existence or coexistence of DCTN1- and/or TDP-43-positive aggregates in individual cells that coexpressed DCTN1-mGFP and mCherry-TDP-43. * *p* < 0.05; ** *p* < 0.01; *** *p* < 0.001; **** *p* < 0.0001 compared to mGFP and mCherry-TDP-43-coexpressing control cells by two-tailed Mann–Whitney test (in (**F**), red asterisks: comparisons based on aggregates of DCTN1 only; blue: comparisons based on aggregates of TDP-43 only; black: comparisons based on both positive aggregates).

We next investigated subcellular localization of disease-linked missense mutants and the truncated mutants of DCTN1, and whether they affected TDP-43 localization in cultured cells (Figure 4, Figure 5 and Figures S2 and S3). Because cytoplasmic aggregates of TDP-43 have been detected in the brains of Perry disease patients, we asked whether expression of Perry disease-linked or truncated mutant DCTN1 caused TDP-43 mislocalization to the outside of the nucleus and aggregation. The mGFP control or DCTN1-mGFP (wild-type or mutants) and mCherry-TDP-43 (wild-type) are transiently transfected into human osteosarcoma U2OS cells, and the cells were cultured under nonstressed conditions. The distribution of fluorescent protein-tagged DCTN1 and TDP-43 in the fixed cells or live cells were imaged by confocal and super-resolution microscopy. This experimental design allowed us to directly observe both DCTN1 and TDP-43 even within aggregates, without performing immunocytochemical staining combined with epitope unmasking.

It has been revealed that, under physiological conditions, TDP-43 undergoes LLPS and dynamically form aggregates in the nucleus [63]. Consistently, in mGFP-coexpressing control cells, mCherry-TDP-43 predominantly resided in the nucleus, and its small aggregates were often observed within the nucleus, confirming that expression of mGFP alone did not affect nuclear localization of TDP-43 (Figure 4A, Figure 5A and Figure S2A). Although overexpression of wild-type DCTN1-mGFP did not affect nuclear localization of the major fractions of TDP-43, moderate but significant increases in TDP-43 aggregation in the nucleus and cytoplasm were detected (Figure 4B, Figure 5A,D–F and Figures S2B and S3A; Movie S1), as compared with mGFP control. On the other hand, expression of DCTN1^G71A^ induced significant levels of cytoplasmic mislocalization and aggregation of TDP-43, partially recapitulating the cellular phenotypes observed in the brains of Perry disease patients (Figure 5). In the cells coexpressing DCTN1^G71A^-mGFP and mCherry-TDP-43, various sized aggregates of both mutant DCTN1 and TDP-43 were distributed to both the cytoplasm and nucleus, and were partially colocalized to each other, as detected by confocal and super-resolution microscopy (Figures S2C,D and S3B; Movie S2). Alternatively, the surfaces of DCTN1 and TDP-43 aggregates appeared to contact each other (Figures S2D and S3C; Movie S3). To our knowledge, this is the first detection of TDP-43 aggregation that was induced by a Perry disease-linked mutant DCTN1 in cultured cells. In contrast, DCTN1^G59S^ and DCTN1^F52L^ mutants induced TDP-43 cytoplasmic aggregation only at low levels, but caused significant levels of TDP-43 nuclear aggregation (Figure 5D–F and Figure S2E,F), thereby reproducing some aspects, especially the severity, of the patient symptoms [50,62]. Quantitative analysis revealed that DCTN1^F52L^, but not DCTN1^G59S^, induced significant mislocalization of TDP-43 into the cytoplasm (Figure 5A). It has been reported that HMN7B-linked DCTN1^G59S^ aggregates are larger and that the aggregate amounts appear to be greater than those of Perry disease-linked mutants [13,47]. Although we confirmed this tendency in cells expressing the DCTN1^G59S^ mutant alone (unpublished observations), less severe levels of DCTN1 protein aggregates were found in cells coexpressing DCTN1^G59S^ and TDP-43, as compared with the cells coexpressing DCTN1^G71A^ and TDP-43 (Figure 5B,C). However, we noted that DCTN1^G59S^ induced statistically significant levels of aggregation of itself and TDP-43 in the nucleus and cytoplasm (Figure 5B–E). Our present observations of DCTN1^F52L^- and TDP-43-coexpressing U2OS cells were also consistent with our previous study using p.F52L-carrying Perry disease patient iPSC-derived tyrosine hydroxylase-positive neurons; in both cell models, aggregates of DCTN1^F52L^, but not TDP-43, were detected in the cytoplasm (Figure 5C,E) [64].

We then examined subcellular localization of truncated mutant DCTN1, and their effects on TDP-43 localization and aggregation. Remarkably, expression of D∆4, D∆5, or D∆6 drastically induced TDP-43 cytoplasmic mislocalization and the aggregation of themselves and TDP-43 in both the cytoplasm and nucleus at significant levels (Figure 4F–H and Figure 5); in particular, we found that phenotypes of D∆4-expressing cells were most similar to those of DCTN1^G71A^, or even exhibited a greater degree of severity in TDP-43 aggregation (Figure 4F, Figure 5 and Figure S2G,H). In such coexpressing cells, D∆4 and TDP-43 showed partial colocalization and their aggregates contacted each other, as in “marble” patterns. Super-resolution live-cell imaging revealed partial colocalization of DCTN1 with TDP-43 and surface-to-surface contacts between their aggregates, as well as segregation of DCTN1 and TDP-43 aggregates in cells coexpressing DCTN1^G71A^ or D∆4 and TDP-43 (Figure S3B–E; Movies S2–S6). Expression of D∆5 led to the formation of large aggregates of TDP-43 in the nucleus (Figure 4G and Figure 5D). D∆6 induced a more severe expression of aggregates of both proteins; coexistence, including colocalization, mutual contacts, and intracellular segregation of DCTN1 and TDP-43 aggregates in either the cytoplasm or the nucleus or both, in the coexpressing cells, was also significantly detected in our quantitative analysis (Figure 4H and Figure 5). In addition, nuclear malformation and fragmentation occurred frequently in those cells coexpressing D∆4, D∆5, or D∆6 and TDP-43 (Figure 4F–H and Figure S2G,H).

In contrast, the D∆1 and D∆2 mutants neither affected localization of the major pools of TDP-43 nor caused TDP-43 cytoplasmic aggregation at detectable levels (Figure 4C,D and Figure 5A,E). However, we found that the appearance of nuclear aggregates of TDP-43 and the incidence of larger nuclear aggregates of TDP-43 were markedly different from those in mGFP control-expressing cells (Figure 4C,D and Figure 5D). This result suggested that CAP-Gly-basic supradomain alone did not affect TDP-43 nuclear localization, but modulated aggregation states in the nucleus, suggesting that intranuclear transport of TDP-43 was impaired. Interestingly, the major fractions of D∆2 were localized predominantly in the nucleus, and to a much lesser extent in the cytoplasm, although D∆1 exhibited a ubiquitous distribution in both the cytoplasm and nucleus (see Discussion). This suggested that the basic domain may have NLS activity, although the major fractions of full-length DCTN1 were located in the cytoplasm, showing microtubular patterns. Overexpression of D∆3, which does not bind to TDP-43, induced low but significant levels of TDP-43 cytoplasmic aggregation (Figure 4E and Figure 5E). Because the DCTN1 CC1 domain interacts with the dynein intermediate chain [5,6], CC1 expression may perturb TDP-43 retrograde transport toward the nucleus. However, TDP-43 cytoplasmic mislocalization was not significantly detected in D∆3-coexpressing cells (Figure 5A).

Taken together, overexpression of several truncated forms of DCTN1 (D∆4–6) prevented the cytoplasmic-nuclear transport of TDP-43, and thereby caused dramatic mislocalization and aggregation of TDP-43, revealing that DCTN1 physiologically regulated TDP-43 localization and aggregation, possibly via multivalent interactions between DCTN1 and TDP-43. The results of expression of truncated DCTN1 (D∆4–6) suggest that the disruption of regulated multivalent DCTN1-TDP-43 interactions drive aberrant aggregation of the two proteins.

### 2.6. Modeling Mutant DCTN1-Induced TDP-43 Aggregation in Human iPSC-Derived Neurons

**Figure 6:**
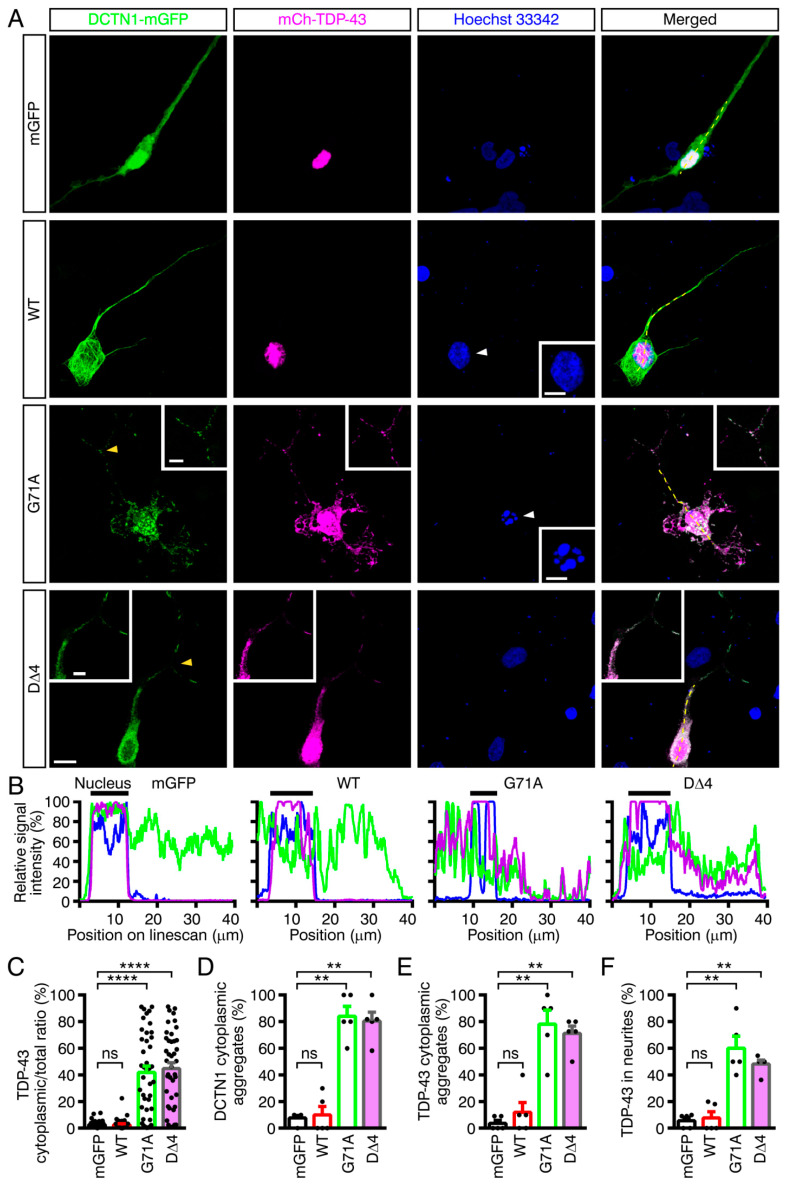
Human iPSC-based modeling of Perry disease. Coexpression of mutant DCTN1 tagged with mGFP and mCherry-tagged wild-type TDP-43 caused cytoplasmic mislocalization and aggregation of TDP-43 in neurons differentiated from *NEUROG2*-inducible human iPSCs. The transfected cells were cultured for two days under nonstressed conditions, fixed, and subjected to confocal microscopy. (**A**) Maximum-intensity projections of deconvoluted z-stack confocal images of neurons coexpressing DCTN1 and TDP-43 constructs as indicated. Note the nuclear malformation (marked with white arrowheads; compare insets in the Hoechst 33,342 panels) in the DCTN1^G71A^ and TDP-43-coexpressing neuron. This phenomenon was often detected in DCTN1^G71A^ or D∆4- and TDP-43-coexpressing neurons. Enlarged images of neurites show the regions marked with yellow arrowheads. Scale bars: 10 μm (large panels); 5 μm, (insets). (**B**) Linescan analyses in (**A**), along the white broken lines. (**C**) Quantification of cytoplasmic mislocalization of TDP-43. *n* = 43, 41, 41, and 42 (neurons) from left to right (four to five experiments). (**D**–**F**) Quantitative analyses of cytoplasmic aggregation of DCTN1 (**D**) and TDP-43 (**E**), and TDP-43 distribution in neurites (**F**). *n* = 5 experiments (10–12 neurons per experimental group). ** *p* < 0.01; **** *p* < 0.0001 compared to mGFP and mCherry-TDP-43-coexpressing control neurons, by two-tailed Mann–Whitney test. ns: not significant.

Human iPSC-derived neuronal cultures have recently been utilized as cellular models of various neurodegenerative diseases [65]. We thus used human iPSC-derived neurons to further recapitulate the aggregation of DCTN1 and TDP-43 in human patient brains. The proneural transcription factor NEUROG2-expressing iPSCs (201B7) were differentiated into neurons harboring neurites (see Section 4.4 for the differentiation procedure) [66,67,68,69,70]. The neurons were transfected with the mGFP control or DCTN1-mGFP (wild-type, p.G71A or D∆4) and mCherry-tagged TDP-43 (wild-type) expression plasmids. The transfected neurons were subjected to confocal microscopy. In cells coexpressing DCTN1^G71A^ or D∆4 with TDP-43, significant levels of TDP-43 cytoplasmic mislocalization were induced (Figure 6A–C). Furthermore, DCTN1 and TDP-43 aggregates of variable sizes were detected in the cytoplasm and neurites (Figure 6A,D,E), revealing that mutant DCTN1 induced mislocalization and aggregation of wild-type TDP-43 protein in human neurons. In particular, to our surprise, ectopic TDP-43 distributions were significantly detected even in neurites of neurons coexpressing DCTN1^G71A^ or D∆4 and TDP-43 (Figure 6A,F). In addition, remarkable levels of clearance of TDP-43 from the nucleus were found in mutant DCTN1-expressing neurons (Figure S4). Furthermore, similar to U2OS cells transfected with mutant DCTN1 and TDP-43, abnormal nuclear morphology was frequently observed in the neurons coexpressing DCTN1^G71A^ or D∆4 and TDP-43 (Figure 6A, p.G71A, inset and Figure S4, D∆4). Taken together with our biochemical finding that DCTN1 binds to TDP-43, these results provide evidence to support a role for DCTN1 in regulating TDP-43 retrograde cytoplasmic-nuclear transport and aggregation in both non-neuronal and neuronal cells. In addition, mutant DCTN1 appeared to cause nuclear membrane disruption.

## 3. Discussion

Although it has long been hypothesized that TDP-43 proteinopathy underlies several neurodegenerative diseases, including ALS, the causal mechanisms remain elusive. The current study revealed that DCTN1 binds to TDP-43. To our knowledge, this is the first demonstration of the biochemical interactions between DCTN1 and TDP-43. *DCTN1* is causative of Perry disease, which has been classified into a type of TDP-43 proteinopathy distinct from ALS/FTD [47,49,50]. TDP-43 proteinopathy and DCTN1 aggregation are two major pathological hallmarks of Perry disease [47,50]. However, the molecular mechanisms by which *DCTN1* mutations cause TDP-43 proteinopathy remained unclear.

Many researchers, including the current authors, have speculated that a direct relationship between DCTN1 aggregation and TDP-43 aggregation was unlikely, and that TDP-43 proteinopathy might be a secondary event that emerges long after dynactin aggregation, mainly for two reasons. First, colocalizations of dynactin components, DCTN1, DCTN2/p50, and DCTN4/p62, with TDP-43 were detected in only a small number of protein inclusions, according to autopsy studies [47,50] and cell-culture studies [71]. Second, dynactin aggregates, but not TDP-43 aggregates, were detected in tyrosine hydroxylase-positive, putative dopaminergic neurons differentiated from Perry disease patient-derived iPSCs (*DCTN1* p.F52L genotype; discussed below) [64].

**Figure 7:**
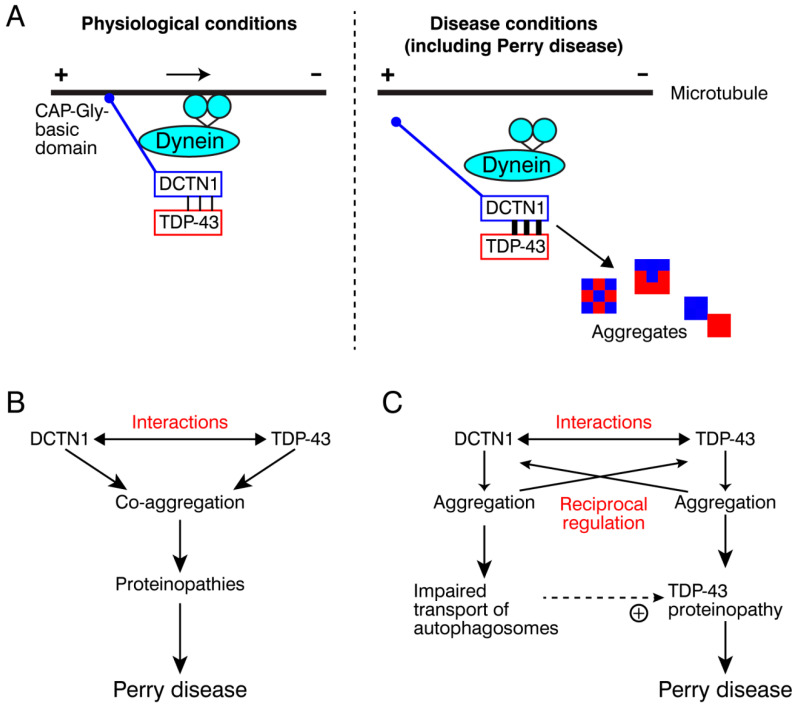
Models for DCTN1 function in TDP-43 cytoplasmic-nuclear transport in health and disease. (**A**) A model for physiological mechanisms of the dynein–dynactin-mediated retrograde transport of TDP-43 along microtubules and pathological mechanisms triggering TDP-43 aggregation in disease conditions, including Perry disease. (**B**,**C**) Two possible, but not mutually exclusive, models of pathological mechanisms that may cause Perry disease (see text).

Our present data revealed that DCTN1 regulates TDP-43 subcellular localization and aggregation, through their interactions, under physiological conditions. Expression of Perry disease-linked missense or truncated forms of mutant DCTN1 induced TDP-43 mislocalization in the cytoplasm and TDP-43 aggregation in the cytoplasm and nucleus. Furthermore, in cultured cells coexpressing mutant DCTN1 (DCTN1^G71A^ or D∆4–6) and wild-type TDP-43, the incidence of aggregation of DCTN1 alone, without TDP-43 aggregation, was unexpectedly low. However, coaggregation (or coexistence within the same cells) of DCTN1 and TDP-43, or less frequently, aggregation of TDP-43 alone, was detected in around 79%–85% of the cells (Figure 5F). It is thus unlikely that DCTN1 aggregation occurs independently of TDP-43 aggregation. Our results suggest that, in Perry disease, TDP-43 aggregates may grow concurrently with those of mutant DCTN1. We consistently found that, in many cells after acute overexpression, aggregates of mutant DCTN1 partially colocalized with those of TDP-43. Alternatively, within the same cells, the surfaces of many aggregates of DCTN1 and TDP-43 frequently appeared to contact each other, according to our observations using confocal and super-resolution microscopy. We also found that mutant DCTN1 remarkably colocalized with TDP-43 in human iPSC-derived neurons in the cytoplasm and axoplasm. Taken together, these results support a model in TDP-43 proteinopathy, including Perry disease (Figure 7A): dysregulation of DCTN1-TDP-43 interactions disrupts dynein-dependent retrograde transport of TDP-43 and causes cytoplasmic mislocalization and aggregation of TDP-43, with concurrent aggregation of DCTN1.

One mechanistic scenario triggering Perry disease is that a missense mutation in the DCTN1 CAP-Gly domain causes dysregulation of DCTN1-TDP-43 interactions, leading to coaggregation of DCTN1 and TDP-43, which drives the symptomatic progression of Perry disease (Figure 7B). The second, but nonmutually exclusive, scenario is that mutant DCTN1 aggregation promotes TDP-43 aggregation, and vice versa, forming reciprocal regulation loops for mutual nucleation (Figure 7C). For example, DCTN1 aggregation may perturb efficient autophagosome transport and cytoplasmic–nuclear transport of TDP-43. Resultant accumulation of TDP-43 aggregates may predominantly contribute to the pathogenesis of Perry disease. To understand the underlying pathogenic mechanisms, further studies are needed to determine the toxicity of DCTN1 aggregates. Alternatively, it is possible that the coexistence of DCTN1 aggregates increases the stability of TDP-43 aggregates, which in turn elevates the toxicity of aggregated TDP-43 [72]. Under physiological conditions, wild-type DCTN1 may suppress cytoplasmic aggregation of TDP-43 by facilitating proper TDP-43 cytoplasmic-nuclear transport and even the nuclear import (see below).

On the other hand, DCTN1 has long been implicated in ALS pathogenesis [73,74,75,76]. A slowly progressive, ALS-like motor neuron disease, HMN7B, is indeed caused by the p.G59S mutation in *DCTN1*, although HMN7B does not accompany TDP-43 proteinopathy at detectable levels [44,50]. In this study, we noted that DCTN1^G59S^ caused moderate but significant levels of TDP-43 aggregation in the nucleus and cytoplasm (Figure 5D,E). In addition, as mentioned above, knockdown of DCTN1 causes motor neuron degeneration [27]. However, the mechanistic basis by which DCTN1 dysfunction contributes to ALS pathogenesis has remained unknown. Our hypothesis that dysregulation of DCTN1–TDP-43 interactions and/or the imbalance in DCTN1 expression perturb TDP-43 transport from axonal tips to the nucleus, and cause TDP-43 aggregation in the cytoplasm and axoplasm may also be applicable to ALS or ALS-related neurodegenerative diseases.

Consistently, several missense mutations in *DCTN1* have been etiologically characterized, such as p.M571T, p.R785W, p.R997W, and p.T1249I, and they reside in the TDP-43-interacting regions [74,75]. These mutation sites and the surrounding regions are evolutionally conserved in vertebrates, with some minor substitutions (Figure S5); such *DCTN1* mutations deserve future investigation.

Thus, our present findings of the interactions between DCTN1and TDP-43, as well as DCTN1 regulation of TDP-43 aggregation, have opened a new avenue toward elucidating not only the physiological mechanisms by which the dynein–dynactin machinery controls TDP-43 transport and distribution but also the pathological mechanisms underlying Perry disease and other TDP-43 proteinopathies, including ALS/FTD. Below we discuss several issues to be addressed in future studies.

### 3.1. DCTN1 Regulates the Cytoplasmic–Nuclear Transport of TDP-43

We found that the expression of Perry disease-linked missense or truncated mutant DCTN1 induces cytoplasmic mislocalization and aggregation of TDP-43, revealing that DCTN1 regulates TDP-43 cytoplasmic-nuclear transport. Cytoplasmic-nuclear transport can be divided into the microtubule-dependent retrograde transport toward the nucleus and the subsequent nuclear import. Because DCTN1 binds to TDP-43, we postulated that the retrograde transport of TDP-43 is mediated by the dynein–dynactin machinery. Furthermore, TDP-43 nuclear import may also be reliant on the dynein–dynactin machinery. Consistent with this idea, it has been shown that transcription factors p53 and NF-κB and the importins are transported to the nucleus in dynein–dynactin-dependent manners [77,78,79]. In addition, it is known that microtubules are tethered to the nuclear pore complex [80]. Therefore, together with the key fact that Perry disease-linked DCTN1 mutants exhibit reduced microtubule-binding abilities [47], our results revealed that DCTN1^G71A^ or truncated mutant DCTN1 disrupts the cytoplasmic-nuclear transport of TDP-43.

Interestingly, CAP-Gly-basic supradomain-containing mutant (D∆2) became localized predominantly in the nucleus (Figure 4D). On the other hand, many other truncated mutants and full-length DCTN1 exhibited ubiquitous, cytoplasmic, or microtubule-associated distributions. We thus hypothesized that the CAP-Gly-basic, especially the basic, domain is able to act as a NLS and that DCTN1 is capable of nucleocytoplasmic shuttling. Supporting these ideas, DCTN1^G71A^ and truncated mutants themselves were often detected in the nucleus, in addition to the cytoplasm (Figure 4 and Figure S2). We therefore propose two possibilities; 1) DCTN1 directly enters the nucleus together with TDP-43 and dynein or 2) DCTN1 delivers TDP-43 to the nuclear import machinery. Related to the latter, the interactions between DCTN1 and importin 7/9 (IPO7/9), similar to importin-β, have already been reported [78,81]. Recent studies have established that importins suppress aggregation of RNA-binding proteins, including Fused in sarcoma (FUS) and TDP-43 [21,82,83,84]. Thus, under physiological conditions, importins and DCTN1 may inhibit TDP-43 aggregation collaboratively before the nuclear import step for TDP-43.

### 3.2. How Does Dynein Interact with the DCTN1-TDP-43 Complex?

mRNA localization underlies precise spatiotemporal control of protein translation in polarized cells, such as neurons, and especially in axons; the dysfunction of this machinery has been implicated in various neurodegenerative diseases [85,86]. The current consensus is that dynein and kinesin motors play roles in mRNA transport and localization [9]. However, it is not fully understood how the motors, especially dynein–dynactin machinery, control mRNA localization in diverse types of mammalian polarized cells. mRNAs are transported after incorporation into cytoplasmic ribonucleoproteins (RNPs). Because TDP-43 is involved in the formation of RNP granules [85,86,87], and because more than 6,000 genes have been identified as RNA targets for TDP-43 [88], the dynein–dynactin machinery may utilize TDP-43 as a cargo adaptor for transporting RNPs. If this is the case, it is possible that TDP-43 recruits the dynein–dynactin machinery to RNPs and that RNP-bound TDP-43 controls dynein motility in collaboration with DCTN1. In this regard, TDP-43 may act as a functional homolog of the *Drosophila* RNA-binding protein Egalitarian (Egl), although *Egl* is not thought to be evolutionally conserved [89]. It remains to be clarified how the other dynactin subunits and dynein associate with the DCTN1-TDP-43 complex. Although dynactin subunits and dynein may compete with TDP-43 for binding sites within DCTN1, how are they are reconciled to build the functional RNP transport machinery? DCTN1 binds to the dynein intermediate chain via the DCTN1 200–811 aa region, which lies across the CC1 and dynactin domains (Figure 1A) [5,6]. We found that dynactin domain also contributes to TDP-43 binding and is capable of inducing TDP-43 aggregation. The C-terminal region of DCTN1 binds to TDP-43, as well as another key dynactin component, Arp1/11 minifilament [1,11]. Thus, it is important to understand how the dynactin domain and C-terminal region balance the interactions with TDP-43 and dynein.

Furthermore, as well as interacting with dynein, DCTN1 interacts with the anterograde microtubule motor kinesins, to coordinate bidirectional transport [14,90]. DCTN1 and dynein (or kinesins) are likely to constitute machineries that drive bidirectional shuttling of TDP-43-containing RNP granules. Dysregulation of multiple interactions among dynein/kinesins, DCTN1, and TDP-43 may perturb RNA transport and cause cytoplasmic mislocalization and aggregation of TDP-43, both of which could contribute to the pathogenesis of Perry disease or other TDP-43 proteinopathies.

### 3.3. Multivalent Interactions between DCTN1 and TDP-43

Electron microscopic studies reveal that the N-terminal region of DCTN1, composed of the CAP-Gly-basic, CC1, dynactin, and CC2 domains form a flexible extension called the projecting arm, while its C-terminal region is buried in the shoulder of the dynactin complex [9,91]. We have characterized the CAP-Gly-basic, dynactin, and CC2 domains and the extreme C-terminal region as TDP-43-interacting sites, revealing that there are multivalent interactions of DCTN1 with TDP-43. Remarkably, TDP-43-interacting sites distribute to the two structural units within DCTN1. However, more detailed analysis of DCTN1-interacting sites of TDP-43, as well as the stoichiometry, is needed to delineate the DCTN1-TDP-43 interactions. Interestingly, such multivalent interactions between different domains of DCTN1 and TDP-43 do not additively contribute to the interactions between full-length DCTN1 and TDP-43 proteins (Figure 2B,D). Although the CAP-Gly-basic supradomain by itself binds to TDP-43, this domain may regulate the TDP-43-interacting activity of the other regions of DCTN1 directly (presumably via intramolecular associations) or indirectly (through efficient microtubule-dependent transport), under physiological conditions.

According to our bioinformatics analysis, five putative IDRs were found in DCTN1. IDRs have been implicated in protein phase separation-driven aggregation [32]. Interestingly, while the CC1 domain has predicted IDRs and the dynactin domain has no detectable IDR, the dynactin domain fragment (D∆4), but not the CC1 fragment (D∆3), notably formed aggregates on its own and induced TDP-43 aggregation (Figure 4E,F and Figure 5). Expression of two C-terminal regions with and without the CC2 domain, corresponding to D∆5 and D∆6, also induced aggregation of themselves and TDP-43. Unexpectedly, in coimmunoprecipitation, D∆7 (corresponding to D∆5 plus D∆6) did not detectably interact with TDP-43 (Figure 2B). Although the underlying mechanism is currently unclear, mutually inhibitory effects on the TDP-43 interaction may be present within the DCTN1 C-terminal region.

Taken together, the present study showed that DCTN1 can form aggregates of DCTN1 and TDP-43, preferentially via the dynactin domain and C-terminal region, which act as TDP-43-binding sites. Our results also implicate biophysical mechanisms by which aggregates of DCTN1 and TDP-43 are formed in Perry-diseased neurons. It remains to be clarified whether non-aggregated forms of mutant DCTN1 triggers TDP-43 aggregation, and whether mutant DCTN1 aggregation occurs earlier than TDP-43 aggregation, or simultaneously. It also remains to be understood whether coaggregation proceeds at the surface or in an intermingled manner or both; interestingly, as mentioned above, we frequently observed these two modes of coaggregation, colocalization and surface-to-surface contacts of DCTN1 aggregates with TDP-43 aggregates, even in the same cells. In addition, our data indicated that the CAP-Gly-basic domain, dynactin domain, and the C-terminal region of DCTN1 act as distinct functional modules for controlling TDP-43 cytoplasmic-nuclear transport and aggregation.

### 3.4. How Does DCTN1 Dysregulation Lead to TDP-43 Aggregation?

Taken together with the previous studies, our results support the two currently well-discussed aspects of Perry disease-linked mutant DCTN1: loss of function (reduced microtubule-binding and impairment of dynactin recruitment at microtubule plus-ends) and gain of function (induction of TDP-43 aggregation), both of which compromise the cytoplasmic-nuclear transport of TDP-43.

Recent studies have revealed that mislocalization of TDP-43 from the nucleus to the cytoplasm and the cytoplasmic aggregation of TDP-43 impairs the machineries for nuclear protein import and RNA export [60,63]. Molecular players involved in nucleocytoplasmic transport have been identified as major components of pathological aggregates of TDP-43. The defects of nucleocytoplasmic shuttling have been proposed to belong to a common mechanism that triggers ALS/FTD and other TDP-43 proteinopathies [60,63,92]. The impairment of the dynein–DCTN1 machinery may be included in such defective transport pathways. As one possible pathological mechanism, dysfunction of the dynein–dynactin machinery may perturb the transport of autophagosomes and aggresomes (to be subjected to autophagy). Such neurodegeneration-evoking mechanisms are likely to be driven by both feedback and feedforward loops and reciprocal regulation, thereby triggering a large deflationary spiral and leading to eventual neurodegeneration.

Consistently, dynein heavy and light chains were found in TDP-43 aggregates purified from transfected neuroblastoma cells [60]. As discussed above, DCTN1 is known to interact with importins. Additionally, altered nuclear morphology, especially nuclear breakdown into small clumps, was frequently observed in DCTN1- and TDP-43-coexpressing cells (Figure 4, Figure 6 and Figure S2), suggesting that DCTN1 and TDP-43 aggregation perturb the integrity and function of the nuclear import machinery, nuclear membrane and nuclear pore complexes. From this view, DCTN1 may be a key TDP-43-binding protein that regulates the nuclear import of TDP-43, possibly collaborating with importins, and the dysfunction of these complexes may trigger mislocalization and aberrant aggregation of TDP-43 in the cytoplasm and nucleus. Different patterns of intranuclear localization of TDP-43 caused by DCTN1 mutants suggest that dynein and DCTN1 play a role in regulating intranuclear transport of TDP-43. Thus, molecular mechanisms that underlie functional coupling of DCTN1 with the nuclear import machinery deserve future investigation.

Nuclear clearance of TDP-43 is another pathological hallmark of TDP-43 proteinopathy [63,92]. Although cytoplasmic mislocalization and aggregation of TDP-43 was reproduced by expressing DCTN1^G71A^, D∆4, or D∆6, nuclear clearance of TDP-43 was observed less frequently (less than 1%) in U2OS cell models coexpressing TDP-43 and mutant DCTN1 (unpublished observations); but significantly (25%–30%) in iPSC-derived neurons coexpressing TDP-43 and mutant DCTN1 (Figure S4). Neuron-specific mechanisms may be involved in the nuclear clearance step of TDP-43 in cells harboring dysfunctional DCTN1. Alternatively, depletion of nuclear pools of TDP-43 may be a time-consuming process, as previously reported [63]. The less efficient retrograde transport may gradually cause TDP-43 nuclear clearance.

### 3.5. Different Effects of Perry Disease- and HMN7B-Linked Mutations on DCTN1 Function

It remains unsolved how closely apposed missense mutations within the DCTN1 CAP-Gly domain (Figure 1A) cause two distinct types of diseases with completely different symptoms: Perry disease that accompanies TDP-43 proteinopathy and HMN7B that does not accompany TDP-43 proteinopathy [50]. We examined the effects of such disease-linked mutations in DCTN1 on TDP-43 interactions and localizations by using our biochemical and cell biological platforms. Perry disease (p.G71A)- or HMN7B (p.G59S)-linked mutations compromised the TDP-43-interacting abilities of DCTN1. While the TDP-43-interacting ability of DCTN1 was elevated by the p.G71A and p.G59S mutations, total amounts of TDP-43 interactions in cells were reduced as compared with wild-type DCTN1 (Figure 3B). On the other hand, drastic aggregation of TDP-43 in the cytoplasm was detected in cells coexpressing TDP-43 and DCTN1^G71A^, but not DCTN1^G59S^, recapitulating some parts of the disease symptoms [50]. Instead, nuclear aggregation of TDP-43 was frequently detected in cells coexpressing DCTN1^G59S^ and TDP-43 (Figure 5D and Figure S2E), suggesting that TDP-43 (micro)aggregation at neuropathologically undetectable levels, especially within the nucleus, may be present in HMN7B patients. Furthermore, colocalizations between DCTN1 and TDP-43 in cells and the presence of their coaggregates support our results that DCTN1^G71A^ and DCTN1^G59S^ retain their TDP-43-interacting ability. Thus, our results do not exclude the possibility that a DCTN1^G59S^-triggered, TDP-43-dependent mechanism contributes to HMN7B pathogenesis, for example, by increasing the instability and degradation of the DCTN1-TDP-43 complex.

In addition to p.G71A, the p.F52L mutation was identified from a causative of Perry disease [62]. In parallel with the finding that the DCTN1^F52L^ mutant exhibited an only slightly reduced microtubule-binding ability, DCTN1^F52L^ retained TDP-43-binding ability at levels comparable with wild-type DCTN1. However, DCTN1^F52L^ significantly induced TDP-43 cytoplasmic mislocalization, in contrast to its slight effects on TDP-43 aggregation, while DCTN1^G71A^ exhibited significant effects on both (Figure 5A,D,E). Thus, Perry disease may be classifiable into subtypes, according to the presence or absence of dramatically formed TDP-43 aggregates. This classification seems to be consistent with the severity of the patient symptoms.

In conclusion, our results show that DCTN1 binds to TDP-43 and regulates the subcellular localization and aggregation of TDP-43 under physiological conditions. We propose a model in which dysregulation of interactions between DCTN1 and TDP-43 causes the cytoplasmic mislocalization and aberrant aggregation of TDP-43 under pathological conditions, including Perry disease. Functional defects in the dynein–dynactin system, especially those in DCTN1, may contribute to the pathogenesis of several TDP-43 proteinopathies, such as ALS.

## 4. Materials and Methods

### 4.1. Protocol Approval in Biological and Animal Experiments

Experimental protocols used in this study were approved by the Institutional Review Boards of Fukuoka University. Animal experiments were performed according to protocols approved by the Institutional Animal Care and Use Committee (IACUC) at Fukuoka University.

### 4.2. Expression Plasmids

cDNA encoding human DCTN1 variant 1 (accession Number: NM_004082) was obtained from Origene (Rockville, MD, USA) (SC110869). The coding region of DCTN1 was amplified by polymerase chain reaction (PCR) and subcloned into pcDNA3.1/CT-GFP-TOPO plasmid (Thermo Fisher Scientific, Waltham, MA, USA). Following confirmation by DNA sequencing, DCTN1 point mutants (p.G71A, p.G59S, or p.F52L) were generated using a QuikChange II XL site-directed mutagenesis kit (Agilent Technologies, Santa Clara, CA, USA). The cDNAs encoding wild-type/full-length DCTN1, point mutants or truncated mutants were reamplified by PCR using KOD Plus (Ver. 2) polymerase (Toyobo, Osaka, Japan), and subcloned into SacII/AgeI sites of mEGFP/pRK5 vector (a gift from K. Svoboda; Addgene, Watertown, MA, USA; #18696), to make DCTN1-mEGFP expression plasmids, or 2× myc/pRK5 (our customized vector; the mEGFP-coding sequence in the mEGFP/pRK5 vector was replaced with 2× myc tag sequence) to make DCTN1-myc expression plasmids.

To clone cDNA encoding human TDP-43, double-stranded Human Liver QUICK-Clone cDNA (Takara-Clontech, Kusatsu, Shiga, Japan) was used for reverse transcription-PCR, according to the manufacturer’s instructions. Full-length cDNAs were cloned into XhoI/BamHI sites of mCherry-C1 plasmid (our customized vector; the mVenus-coding sequence in mVenus-C1 vector (a gift from A. Miyawaki; Addgene; #54651) was replaced with the mCherry-coding sequence). mCherry-TDP-43-∆NLS was generated using a QuikChange Lightning site-directed mutagenesis kit (Agilent Technologies), based on the same design as previously described [20]. The coding sequences of the NTF and CTF of TDP-43 were reamplified by PCR and subcloned into the mCherry-C1 vector. For bacterial expression, TDP-43-coding sequence was subcloned into BamHI/XhoI sites of pGEX6P-1 vector (Cytiva, Tokyo, Japan).

The nucleotide sequences of DCTN1, TDP-43, their missense mutants and truncated mutants used in this study were confirmed by DNA sequencing (Fasmac, Atsugi, Kanagawa, Japan).

### 4.3. Antibodies

Antibodies against DCTN1 (goat polyclonal; Abcam, Cambridge, UK; ab11806), TDP-43 (against the C-terminal region; rabbit polyclonal; Proteintech; #12892-1-AP), and β-tubulin (mouse monoclonal (mAb); Sigma-Aldrich, St. Louis, MO, USA; TUB2.1) were used for immunoprecipitation and/or Western blotting. Antibodies against GFP (polyclonal; MBL; #598), RFP (mouse monoclonal cocktail; MBL; M208-3), c-Myc (mouse monoclonal used for immunoprecipitation; Santa Cruz Biotechnology, Santa Cruz, CA, USA; 9E10), Myc-tag (rabbit polyclonal used for Western blotting; MBL; #562), and horseradish peroxidase (HRP)-conjugated anti-RFP antibody (MBL; PM005-7) were also used for the detection of tagged DCTN1 and TDP-43 proteins.

HRP-conjugated secondary antibodies used for Western blotting in this study were: goat antimouse IgG (H + L; Bio-Rad, Hercules, CA, USA; #1706516), AffiniPure donkey antigoat IgG (Jackson ImmunoResearch, West Grove, PA, USA; #705-035-147), Amersham ECL donkey antirabbit IgG (Cytiva; NA934), and mouse antirabbit IgG (conformation-specific) mAb (Cell Signaling Technology, Danvers, MA, USA; L27A9; #5127).

### 4.4. Cell Culture and Transfection

African green monkey (*Cercopithecus aethiops*) fibroblast COS-7 cells and human osteosarcoma U2OS cells were obtained from ATCC. Cells were maintained in Dulbecco’s modified Eagle’s Medium (DMEM; Thermo Fisher Scientific, #11965092) supplemented with 10% fetal bovine serum (FBS; from HyClone/Cytiva, Tokyo, Japan), 1% penicillin/streptomycin (Thermo Fisher Scientific), and 2 mM L-glutamine (Thermo Fisher Scientific). All cells were cultured in a 5% CO_2_ incubator at 37 °C.

For transfection with plasmids, cells were seeded at 2.0 × 10^5^ cells per well in a six-well plate (Falcon/Corning, Corning, NY, USA), without any antibiotic treatment, ~24 h before transfection. Transient transfection was performed using Lipofectamine 3000 (Thermo Fisher Scientific), according to the manufacturer’s instructions. Briefly, cells were transfected in Opti-MEM I reduced serum medium (Thermo Fisher Scientific; #31985062) by adding a mixture of 6 μL of Lipofectamine 3000 reagent in Opti-MEM and combinations of plasmids plus 5 μL of P3000 reagent in Opti-MEM I [93]. The following amounts of DNA (per well) were used for transfection: 500 ng DCTN1-mGFP (mGFP or DCTN1-myc) and 300 ng mCherry-TDP-43. Cells were incubated in the presence of the transfection mixture for 5 h, washed once, and then their culture medium was replaced. The transfected COS-7 were cultured for 48 h for immunoprecipitation. For microscopy, one day later, the U2OS cells were replated onto fibronectin (Thermo Fisher Scientific)-coated coverslips (diameter: 12 mm; Matsunami Glass, Kishiwada, Osaka, Japan) or glass-based culture dishes (Iwaki, AGC Techno Glass; Haibara, Shizuoka, Japan). For microscopic observation, the cells were cultured for a total of 60–72 h after transfection and fixed by the addition of 4% PFA + 10% sucrose in phosphate-buffered saline (PBS), which was equilibrated to room temperature, directly to the culture. The fixed cells were treated with 50 mM NH_4_Cl for 10 min at room temperature to reduce autofluorescence, and incubated with Hoechst 33,342 (Molecular Probes/Thermo Fisher Scientific) for nuclear counterstaining. Samples were mounted in Permafluor (Thermo Fisher Scientific), and observed under a confocal microscope.

*NEUROG2*-inducible human iPSC (201B7) lines, where NEUROG2 is overexpressed upon treatment with doxycycline, were maintained using a feeder-free culture system (StemFit; Ajinomoto, Tokyo, Japan/Takara; AK02N) [69,70]. The iPSCs (1.0 × 10^5^ cells/well) were replated onto poly-D-lysine/laminin-coated coverslips (BioCoat/Corning; #354087) in a 24-well plate or μ-dishes (ibidi, Lochhamer Schlag, Gräfelfing, Germany; ib81156), and directly differentiated into neurons by treatment with 1 μg/mL doxycycline for 7 days and then by stimulation with 20 ng/mL brain-derived neurotrophic factor (BDNF; Peprotech, Cranbury, NJ, USA; #450-02), 20 ng/mL Glial cell line-derived neurotrophic factor (GDNF; Peprotech; #450-10), 1mM dibutyryl cyclic AMP (Sigma-Aldrich; D0260), and 200 nM ascorbic acid (Sigma; A5960) in Neurobasal medium (Thermo Fisher Scientific; #21103-049) supplemented with B-27 (Thermo Fisher Scientific; #17504-044) for 3 days. After a total of 10 days of neuronal differentiation, iPSC-derived neurons were cotransfected with the DCTN1-mGFP (wild-type, p.G71A, D∆4, D∆5 or D∆6) and mCherry-TDP-43 expression plasmids using Lipofectamine 3000, following the same protocol as above. The cells were fixed 48 h after transfection by adding 4% PFA + 10% sucrose in PBS, stained with Hoechst 33,342 and observed under a confocal microscope. In the current experiments, the survival rates of neurons coexpressing D∆5 or D∆6 and TDP-43 were too low for observation, possibly due to their cell death-inducing effects in differentiated neurons. Thus, analyses on these cells were excluded from our present study.

### 4.5. Coimmunoprecipitation, GST Pull-Down, and Western Blotting

For coimmunoprecipitation between endogenous Dctn1 and Tdp-43 proteins, cell extracts were prepared from embryonic day 16.5 (E16.5) mouse whole-brain isolated from pregnant ICR mice (SLC, Hamamatsu, Shizuoka, Japan). The buffer used contained 50 mM Tris-HCl (pH7.4), 150 mM sodium chloride, 1 mM EDTA, 0.5% NP-40, and 0.5% sodium deoxycholate; supplemented with 5 mM sodium orthovanadate, 10 mM sodium fluoride, and cOmplete Mini (Merck; #11836153001). The brain cell lysates were preabsorbed with Protein G-conjugated Sepharose (Cytiva), and then incubated with a goat anti- DCTN1 antibody at 4 °C for 17 h. The immunoprecipitates were recovered using SureBeads Protein G magnetic beads (Bio-Rad; #1614023) for 2 h. Beads were collected by using DynaMag-2 (Thermo Fisher Scientific) and washed five times with the above buffer. Recovered proteins were eluted by adding 1 × Laemmli buffer to the beads and boiled at 95 °C for 5 min. The proteins were detected by Western blotting with the antibodies indicated in the Figure 1, Figure 2 and Figure 3.

For coimmunoprecipitation of tagged proteins, COS-7 cells were transfected with different combinations of plasmids encoding the wild-type/full-length, truncated mutant or point mutant protein of DCTN1 or TDP-43. Forty-eight hours after transfection, cell lysates were prepared using the same buffer as above except without sodium deoxycholate, and immunoprecipitated with anti-c-Myc, anti-GFP or anti-RFP antibodies and then with SureBeads Protein G magnetic beads.

To detect the in vitro binding between DCTN1 and TDP-43, recombinant GST-fused full-length TDP-43 proteins were expressed in *E.coli* BL21-CodonPlus (DE3)-RIL (Agilent Technologies) at 15.5 °C for 16 h upon induction with 100 mM IPTG [31]. After bacterial lysis in BugBuster Master Mix (Merck, Darmstadt, Hessen, Germany) supplemented with EDTA-free cOmplete Mini (Merck), GST or GST-TDP-43 proteins were purified using glutathione Sepharose 4B beads (Cytiva) in PBS supplemented with 1 mM DTT and EDTA-free cOmplete Mini. Lysates from cells expressing D∆8 and D∆9-mGFP were prepared in a buffer containing 50 mM Tris-HCl (pH7.4), 100 mM sodium chloride, 3mM magnesium chloride, 10% glycerol, 1% Triton X-100, and 1 mM DTT, supplemented with 5 mM sodium orthovanadate, 10 mM sodium fluoride, and EDTA-free cOmplete Mini. The cell lysates were incubated with GST or GST-TDP-43-preloaded glutathione beads at 4 °C for 2 h. After washing with a buffer containing 50 mM Tris-HCl (pH 7.4), 100 mM sodium chloride, 3mM magnesium chloride, 10% glycerol, 1% NP-40, and 1 mM DTT, supplemented with EDTA-free cOmplete Mini, the bound proteins were eluted by adding 1× Laemmli buffer to the beads.

Immunoprecipitates, pull-down samples, and cell lysates were resolved on an SDS-PAGE gel (home-made or e-PAGEL, Atto, Japan) together with Precision plus protein dual color standards (Bio-Rad), transferred onto Immobilon-P PVDF membranes (Millipore), and probed with the primary antibody as indicated and HRP-conjugated secondary antibodies as previously described [93]. Immunoreactive signals were detected using Amersham ECL Prime Western blotting detection reagent (Cytiva) on an Amersham Imager 680 (Cytiva). Quantification of signal intensity on the Western blots (Figure 3) was performed using ImageJ/Fiji (1.52p; Gel Analyzer tool) software. Each of the biochemical experiments were performed 2–5 times and representative results are shown in Figure 1, Figure 2 and Figure 3.

### 4.6. Microscopy and Quantification

Images of single optical sections and/or z-stacks of fixed cells were acquired using a laser-scanning confocal LSM710 microscope or super-resolution LSM880Airy microscope (in confocal mode) (Carl Zeiss, Jena, Thüringen, Germany). Quantification of images was performed using ZEN 3.0 SR (black) (Carl Zeiss) and MetaMorph (version 7.10; Molecular Devices, San José, CA, USA). Images presented in Figure 4, Figure 6 and Figures S2 and S4 were deconvoluted on MetaMorph and merged using Photoshop (Adobe, San José, CA, USA) for data presentation. Linescan analyses were performed using MetaMorph. Scale bars were prepared using ZEN 3.0 SR and MetaMorph.

The cells to be quantified were randomly selected on each coverslip. In each experiment shown in Figure 4 and Figure 5, ~50 cells (accurately, 50–60 cells) per experimental group were imaged using the LSM710 microscope. For the quantitative analyses shown in Figure 5A and Figure 6C, thresholds were set at 3.5-fold background in the randomly chosen cell images (from three to five experiments). Integrated signal intensity of mCherry-TDP-43 within the traced region (the perimeter of the nucleus and cell) were calculated using MetaMorph. For tracing iPSC-derived neurons, the region composed of the cell body and all major neurites (30 μm-length proximal parts (the main neuritic shafts) from the cell body) was subjected to quantification. The ratio of (total intensity-nuclear intensity) to total intensity was presented as the TDP-43 cytoplasmic/total ratio (percent) in Figure 5A and Figure 6C. In the quantitative analyses shown in Figure 5B–F, cells with three or more DCTN1- or-TDP-43-positive aggregates in the nucleus or cytoplasm were counted as cells with nuclear or cytoplasmic-localized DCTN1- or TDP-43-positive aggregates. The aggregates were identified by mEGFP- or mCherry-positive accumulations in the nucleus or cytoplasm in the acquired confocal images. Cells with three or more aggregates of DCTN1 or TDP-43 in the cytoplasm (alternatively, one or more large aggregate with a diameter of 4 μm or more) were regarded as cells with cytoplasmic aggregates. In U2OS cells, even in nontransfected and control cells, mCherry-TDP-43 tended to form aggregates in the nucleus; therefore, cells having three or more nuclear aggregates of TDP-43 with a diameter of 2 μm or more (alternatively, one or more nuclear aggregate with a diameter of 4 μm or more) were referred to as cells with nuclear aggregates. Quantification was performed primarily by one investigator (M.K.-K.), and the results were validated by a different investigator (J.Y.-K.). The percentage of DCTN1 and/or TDP-43 aggregate-carrying cells was calculated ((number of cells with aggregates/number of cotransfected cells examined) × 100 for each experimental group). The results of six (mGFP control) or three (the other groups) independent experiments in U2OS cells were presented. The same quantitative strategy was applied to the experiments in iPSC-derived neurons (Figure 6D–F), except that ~10 cells per experimental group were imaged using the LSM710 or LSM880Airy microscope in confocal mode, and subsequently analyzed. In Figure S4, cells displaying nuclear clearance of TDP-43 were defined as those in which the average signal intensity of mCherry-TDP-43 in the cytoplasm was higher than in the nucleus.

Live-cell microscopy was performed using the LSM880Airy microscope (in super-resolution mode) with a 63×/NA 1.40 oil objective lens (Carl Zeiss) and an Incubation System S (PeCon GmbH, Erbach, Hessen, Germany) at 37 °C and in a 5% CO_2_ atmosphere. Approximately 48–72 h after transfection, cells were live-imaged in CO_2_-dependent FluoroBrite DMEM media (Thermo Fisher Scientific), supplemented with 10% FBS, 1% penicillin/streptomycin, and 2 mM L-glutamine. Super-resolution images were acquired at intervals between approximately 4 and 6 s, in a single focal plane.

### 4.7. Statistical Analysis

No statistical methods were used to predetermine sample size. Sample sizes were chosen based on previous experience to obtain reproducibility. The experiments were not randomized and the investigators were not blinded to allocation during experiments and outcome assessment. No data points were excluded, and all data collected from each individual experiment were used for analysis. Statistical analysis was performed using the two-tailed paired *t*-test or two-tailed Mann–Whitney test (Prism 6.04 software, GraphPad, San Diego, CA, USA); *p* < 0.05 was considered statistically significant.

## Data Availability

The datasets generated and analyzed during the present study are available from the corresponding authors on reasonable request.

## Supplementary Materials

The following are available online at https://www.mdpi.com/article/10.3390/ijms22083985/s1, Figure S1: Bioinformatics analyses on DCTN1 and TDP43; Figure S2: Effects of missense mutant DCTN1 and D∆4 on TDP-43 mislocalization and aggregation; Figure S3: Super-resolution live-cell imaging of dynamics of wild-type (WT) or mutant DCTN1-mGFP (p.G71A or D∆4) and mCherry-TDP-43 (WT) in U2OS cells; Figure S4: Nuclear clearance of TDP-43 in iPSC-derived neurons co-expressing mutant DCTN1 and wild-type TDP-43; Figure S5: Amino acid sequence conservation in the dynactin domain and C-terminal region of DCTN1 across several animal species. Movie S1: A representative case of super-resolution live-cell imaging of a U2OS cell co-expressing DCTN1-mGFP (wild-type [WT]) and mCherry-TDP-43 (WT); Movie S2: Super-resolution live-cell imaging of a U2OS cell co-expressing DCTN1-mGFP (p.G71A) and mCherry-TDP-43 (WT); Movie S3: Super-resolution live-cell imaging of a U2OS cell co-expressing DCTN1-mGFP (p.G71A) and mCherry-TDP-43 (WT); Movie S4: Super-resolution live-cell imaging of a U2OS cell co-expressing DCTN1-mGFP (D∆4) and mCherry-TDP-43 (WT); Movie S5: Super-resolution live-cell imaging of a U2OS cell co-expressing DCTN1-mGFP (D∆4) and mCherry-TDP-43 (WT); Movie S6. Super-resolution live-cell imaging of a U2OS cell co-expressing DCTN1-mGFP (D∆4) and mCherry-TDP-43 (WT).

## Abbreviations

ALS	Amyotrophic lateral sclerosis
CAP-Gly	Cytoskeleton-associated protein glycine-rich
CC	Coiled-coil
*DCTN1*	Dynactin subunit 1
FTD	Frontotemporal dementia
GFP	Green fluorescent protein
GST	Glutathione-S-transferase
HMN7B	Distal hereditary motor neuropathy 7B
IDR	Intrinsically disordered region
IP	Immunoprecipitation
iPSC	Induced pluripotent stem cell
LLPS	Liquid-liquid phase separation
MT	Microtubule
NCPR	Net charge per residue
NES	Nuclear export signal
NLS	Nuclear localization signal
PCR	Polymerase chain reaction
PONDR	Predictor of naturally disordered regions
PrLD	Prion-like domain
RFP	Red fluorescent protein
RNP	Ribonucleoprotein
TCL	Total cell lysates
TDP-43	TAR DNA-binding protein 43
