# Supplementary material for: DCTN1 Binds to TDP-43 and Regulates TDP-43 Aggregation
Source: Int J Mol Sci. 2021 Apr 13;22(8):3985. doi: 10.3390/ijms22083985 (PMC8070438; doi:10.3390/ijms22083985)
Supplement: Supplementary file 1 [file ijms-22-03985-s001.zip › ijms-1172969-SupMaterials-041221-2F.pdf]

Supplementary Materials

DCTN1 binds to TDP-43 and regulates TDP-43 aggregation

Manami Deshimaru, Mariko Kinoshita-Kawada, Kaori Kubota, Takuya Watanabe,

Yasuyoshi Tanaka, Saito Hirano, Fumiyoshi Ishidate, Masaki Hiramoto, Mitsuru Ishikawa,

Yoshinari Uehara, Hideyuki Okano, Shinichi Hirose, Shinsuke Fujioka,

Katsunori Iwasaki, Junichi Yuasa-Kawada, Takayasu Mishima and Yoshio Tsuboi

Supplementary data inventory

Figure S1

Figure S2

Figure S3

Figure S4

Figure S5

Movie S1

Movie S2

Movie S3

Movie S4

Movie S5

Movie S6

Original blot and gel images (Supporting Information)

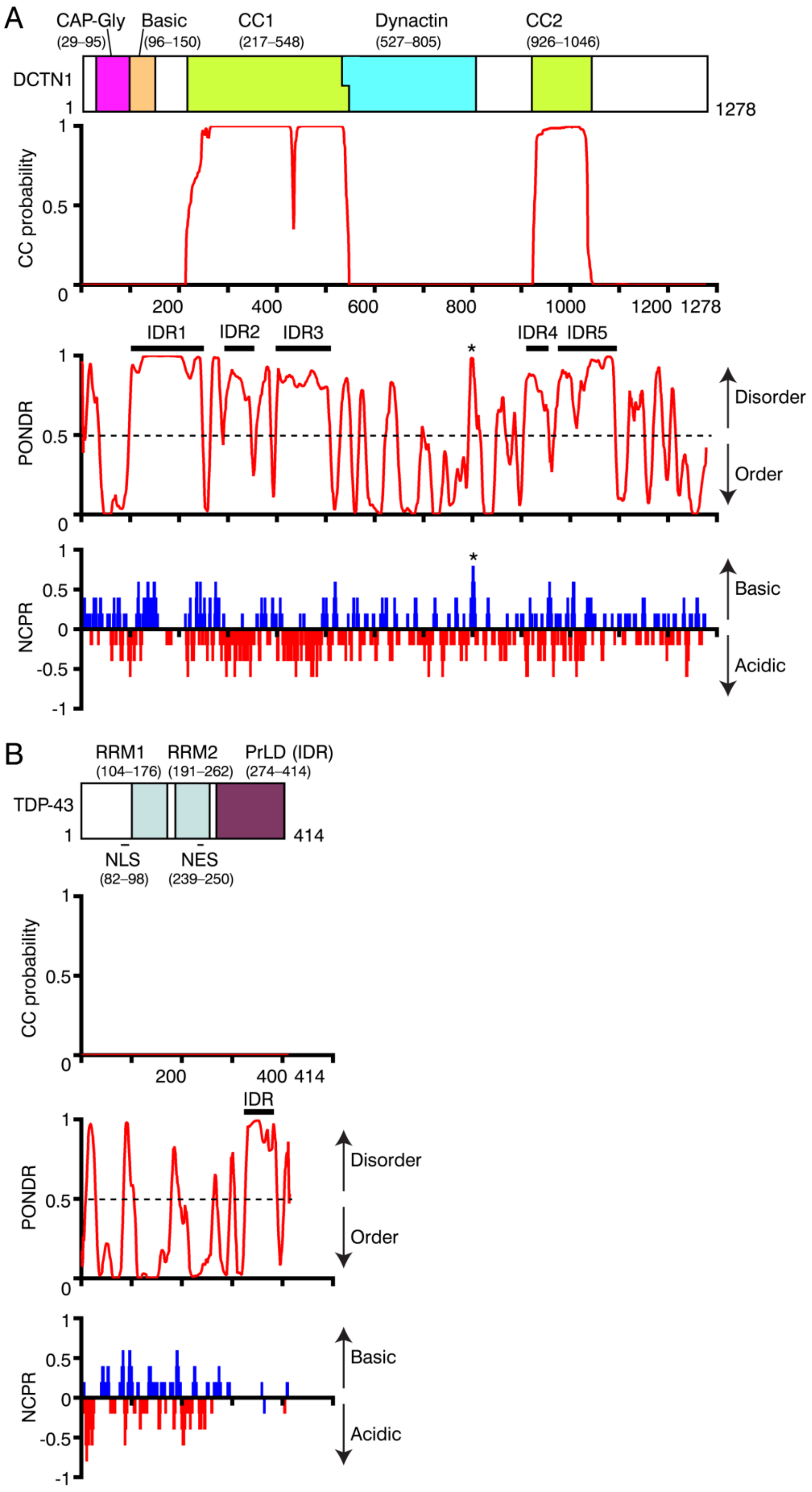
A
CAP-Gly
(29–95)
Basic
(96–150)
CC1
(217–548)
Dynactin
(527–805)
CC2
(926–1046)
DCTN1
1
1278
CC probability
200
400
600
800
1000
1200
IDR1
IDR2
IDR3
IDR4
IDR5
PONDR
Disorder
Order
NCPR
Basic
Acidic
B
RRM1
(104–176)
RRM2
(191–262)
PrLD (IDR)
(274–414)
TDP-43
414
NLS
(82–98)
NES
(239–250)
IDR

Figure S1. Bioinformatics analyses on DCTN1 and TDP43. (**A**) Analyses on DCTN1. Results of coiled-coil (CC) prediction by Multicoil2, disordered domain prediction by PONDR (predictor of natural disordered regions), and NCPR (net charge per residue; 5-amino acid window) analyses are shown. Black bars in the PONDR graph show predicted intrinsically disordered regions (IDRs). In NCPR, charged residues are shown in blue (basic: Arg and Lys) and red (acidic: Asp and Glu). (**B**) Analyses on TDP-43. Results of CC prediction, PONDR, and NCPR are presented.

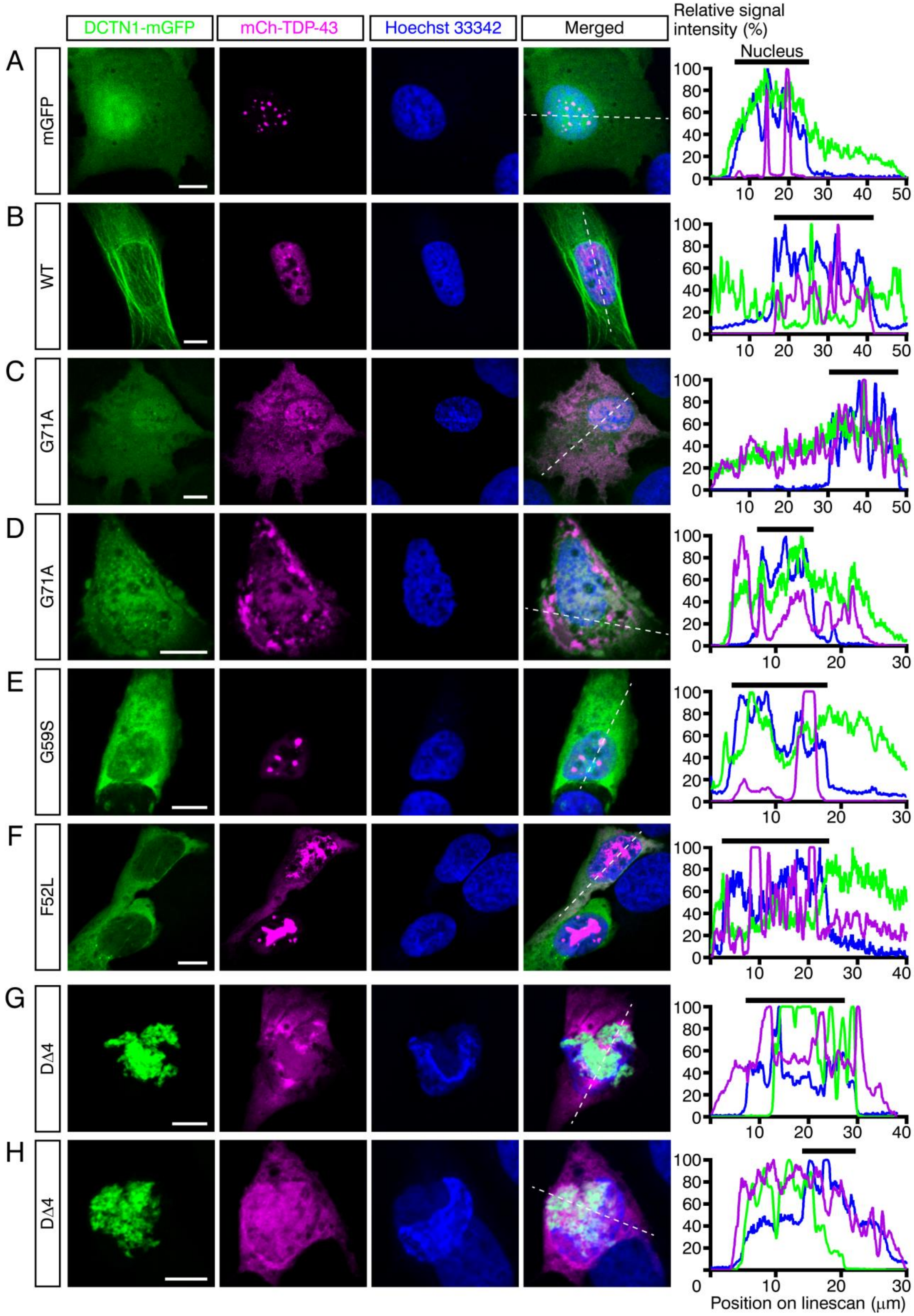

DCTN1-mGFP
mCh-TDP-43
Hoechst 33342
Merged
Relative signal intensity (%)
Nucleus
A
mGFP
B
WT
C
G71A
D
G71A
E
G59S
F
F52L
G
DΔ4
H
DΔ4
Position on linescan (μm)

Figure S2. Effects of missense mutant DCTN1 and DΔ4 on TDP-43 mislocalization and aggregation. Maximum-intensity projections of deconvoluted z-stack confocal images of U2OS cells that co-expressed mGFP (**A**), wild-type (WT) (**B**) or mutant (**C-H**) DCTN1-mGFP and mCherry-tagged WT TDP-43 are shown. The transfected cells were cultured under non-stressed conditions, fixed three days after transfection, and subjected to confocal microscopy. In (F), two representative cellular phenotypes of DCTN1^{F52L}-mGFP- and mCherry-TDP-43-coexpressing cells are shown: nuclear aggregation and ubiquitous cytoplasmic distribution of TDP-43. Scale bars, 10 μm. The right graphs show linescans of the cells along the white broken lines.

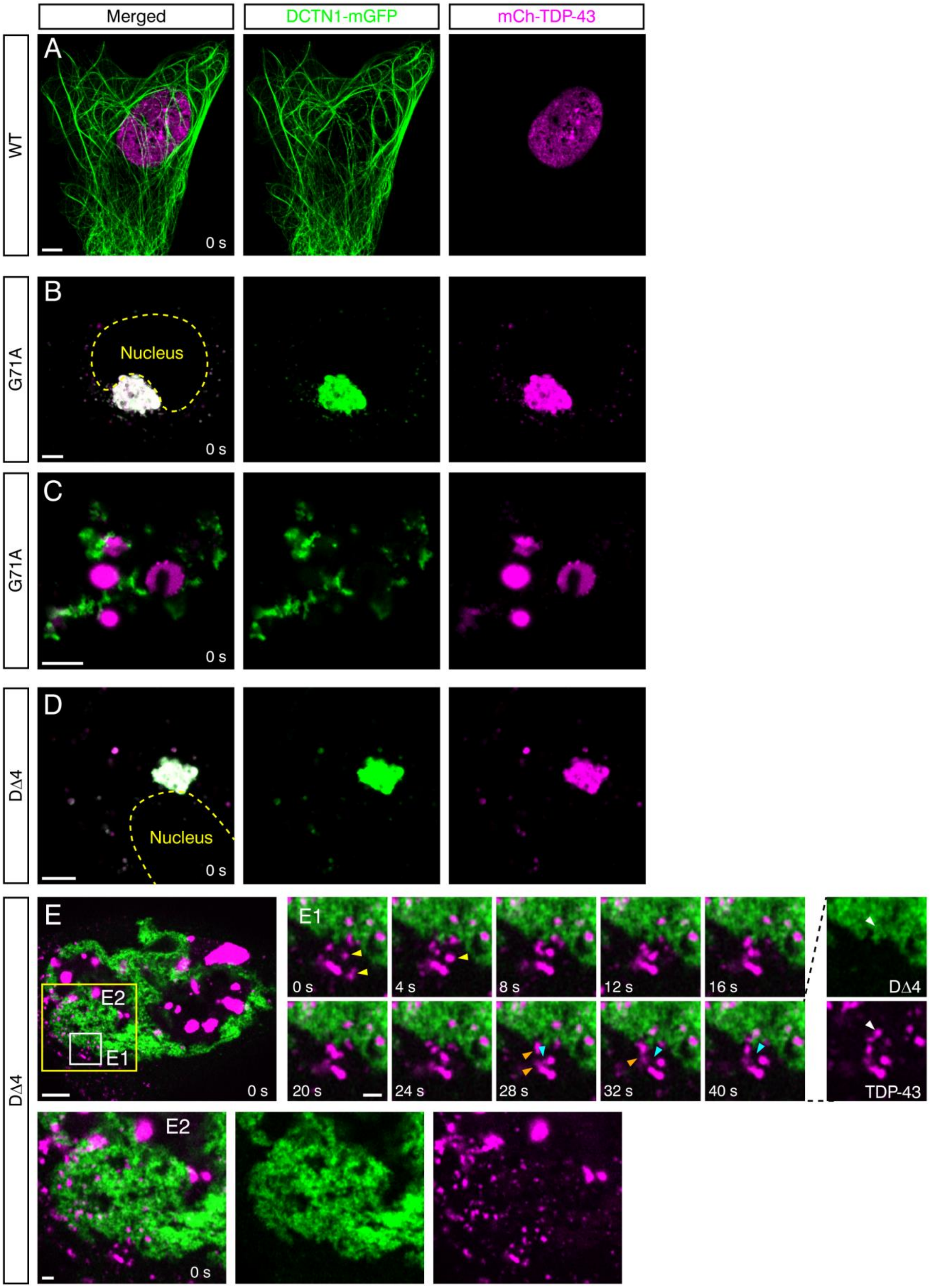
Merged
DCTN1-mGFP
mCh-TDP-43
WT
A
0 s
B
G71A
Nucleus
0 s
C
G71A
0 s
D
DΔ4
Nucleus
0 s
E
DΔ4
E2
E1
0 s
E1
0 s
4 s
8 s
12 s
16 s
20 s
24 s
28 s
32 s
40 s
DΔ4
TDP-43
E2
0 s

Figure S3. Super-resolution live-cell imaging of dynamics of wild-type (WT) or mutant DCTN1-mGFP (p.G71A or DΔ4) and mCherry-TDP-43 (WT) in U2OS cells. (**A**) A snap shot of U2OS cells co-expressing WT DCTN1-mGFP (shown in green) and mCherry-TDP-43 (WT; purple). (**B,C**) Snap shots of aggregates of mutant DCTN1 (p.G71A)-mGFP and mCherry-TDP-43 (WT). In (C), the nucleus is located above the top-left corner. (**D,E**) Snap shots of aggregates of mutant DCTN1 (DΔ4)-mGFP and mCherry-TDP-43 (WT). E1: time-lapse imaging of the white-boxed area in (E) for 40 s. mCherry- TDP-43 aggregates dynamically undergo remodeling through repeated contacts among neighboring aggregates of DCTN1 (DΔ4)-mGFP and mCherry-TDP-43, as well as fission/fusion cycles. Two fusion processes between mCherry-TDP-43 aggregates are marked with yellow and orange arrowheads, while a fission process of mCherry-TDP-43 aggregates is marked with cyan arrowheads (before and after the event). An obviously found colocalization of DCTN1 with TDP-43 is marked by white arrowheads (the right panels). E2: High-power views of the yellow-boxed area in (E). Scale bars, 5 μm (A-E) and 1 μm (E1 and E2). See Movie S1–S6.

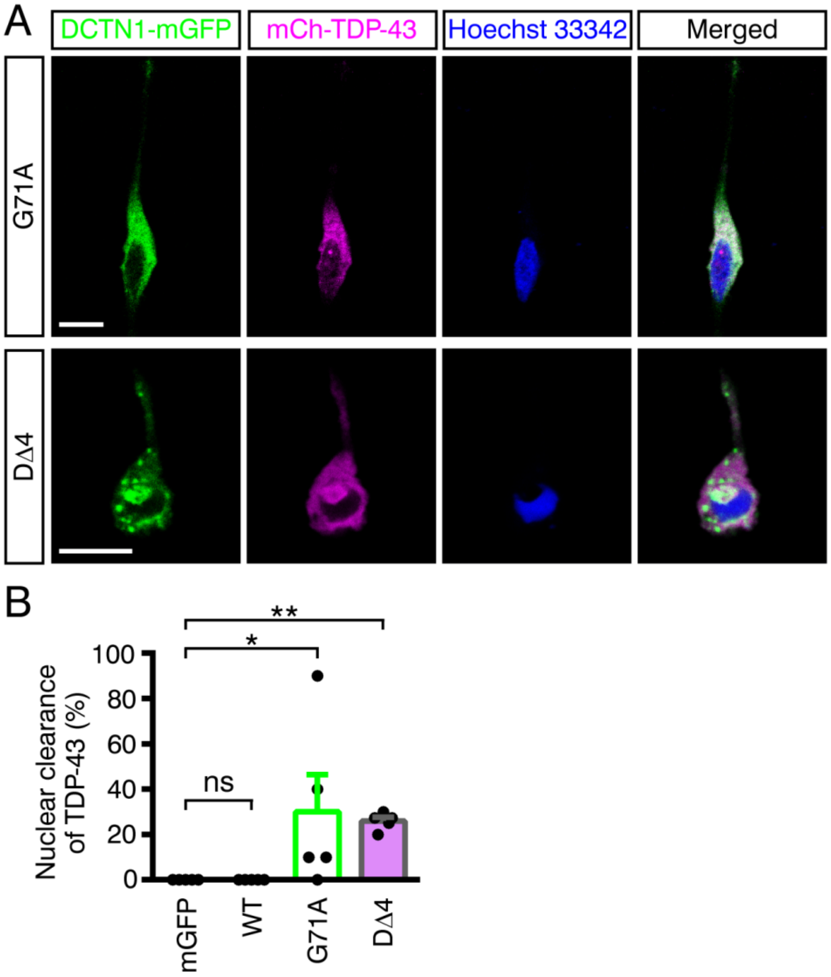

Figure S4. Nuclear clearance of TDP-43 in iPSC-derived neurons co-expressing mutant DCTN1 and wild-type TDP-43. (**A**) Examples of nuclear clearance of mCherry-TDP-43 in mutant DCTN1-mGFP (p.G71A or DΔ4)-coexpressing neurons. Single-section confocal images of deconvoluted z-stacks are shown. Scale bars, 10 μm. (**B**) Quantitative analysis of nuclear clearance of TDP-43. n = 5 experiments (10–12 cells per experimental group). * $p < 0.05$; ** $p < 0.01$ by two-tailed Mann-Whitney test. ns: not significant.

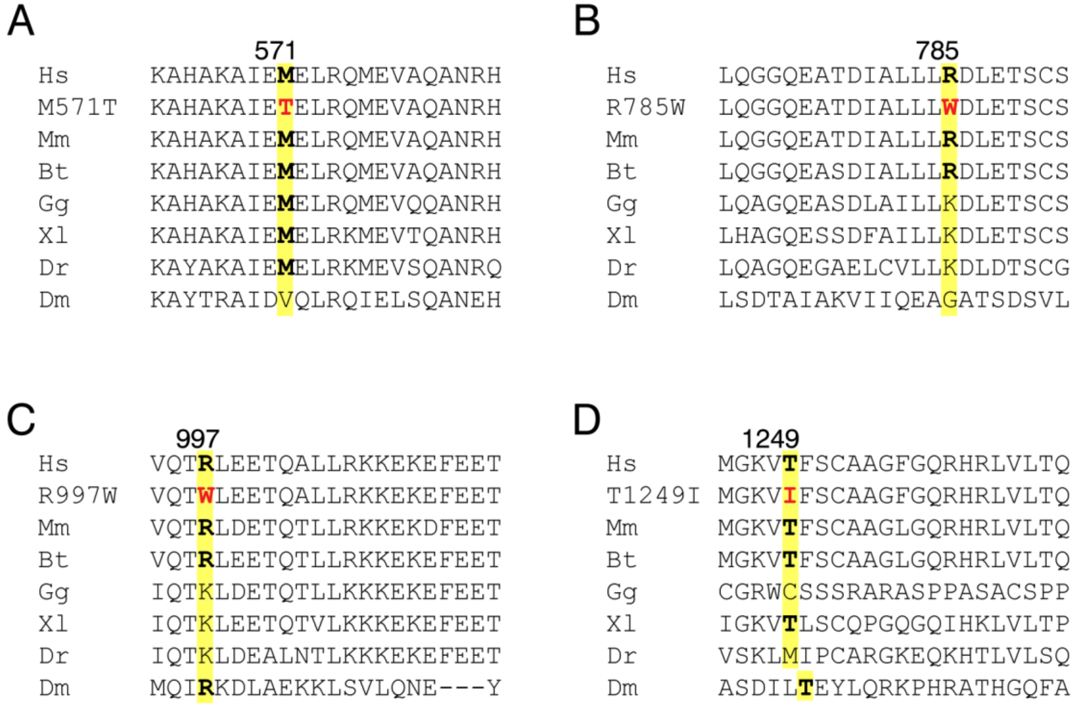

Figure S5. Amino acid sequence conservation in the dynactin domain and C-terminal region of DCTN1 across several animal species. (**A-D**) Four segregated sites of missense mutations from patients with ALS are shown; (A) p.M571T, (B) p.R785W, (C) p.R997W, and (D) p.T1249I. The positions of putative ALS-linked mutations are highlighted in bold. Hs: *Homo sapiens*; Mm: *Mus musculus*; Bt: *Bos Taurus*; Gg: *Gallus gallus*; Xl: *Xenopus laevis*; *Dr: Danio rerio*; Dm: *Drosophila melanogaster*.

Movie S1. A representative case of super-resolution live-cell imaging of a U2OS cell co-expressing DCTN1-mGFP (wild-type [WT]; shown in green) and mCherry-TDP-43 (WT; purple). The example is from Figure S3A. Images were taken every 6 s, and are shown at 3 frames per second.

Movie S2. Super-resolution live-cell imaging of a U2OS cell co-expressing DCTN1-mGFP (p.G71A; shown in green) and mCherry-TDP-43 (WT; purple). The example is from Figure S3B. A large cytoplasmic aggregate, near the nucleus, in which DCTN1^{G71A}-mGFP and mCherry-TDP-43 colocalized, was found. Images were taken every 6 s, and are shown at 3 frames per second.

Movie S3. Super-resolution live-cell imaging of a U2OS cell co-expressing DCTN1-mGFP (p.G71A; shown in green) and mCherry-TDP-43 (WT; purple). The example is from Figure S3C. Cytoplasmic DCTN1^{G71A} aggregates partially colocalized with or repeatedly contacted those of TDP-43. Images were taken every 6 s, and are shown at 3 frames per second.

Movie S4. Super-resolution live-cell imaging of a U2OS cell co-expressing DCTN1-mGFP (DΔ4; shown in green) and mCherry-TDP-43 (WT; purple). The example is from Figure

S3D. A large cytoplasmic aggregate in which DΔ4 and TDP-43 colocalized was observed. Images were taken every 4 s, and are shown at 3 frames per second.

Movie S5. Super-resolution live-cell imaging of a U2OS cell co-expressing DCTN1-mGFP (DΔ4; shown in green) and mCherry-TDP-43 (WT; purple). A whole-cell view; the same cells as that shown in Figure S3E was live-imaged. Aggregates of mCherry-TDP-43 interacted with massive aggregates of DΔ4-mGFP. Images were taken every 4 s, and are shown at 3 frames per second.

Movie S6. Super-resolution live-cell imaging of a U2OS cell co-expressing DCTN1-mGFP (DΔ4; shown in green) and mCherry-TDP-43 (WT; purple). A higher-power view of the cell presented in Movie S5. Microaggregates of mCherry-TDP-43 repeatedly contacted the aggregates of neighboring aggregates of mCherry-TDP-43 and those of DCTN1 (DΔ4), and underwent cycles of fission and fusion, via a liquid-liquid phase separation (LLPS)-mediated process. mCherry-TDP-43 aggregates frequently appeared to grow in such a manner. Images were taken every 4 s, and are shown at 1.5 frames per second. See Figure S3E (E1).
